# Magnetar emergence in a peculiar gamma-ray burst from a compact star merger

**DOI:** 10.1093/nsr/nwae401

**Published:** 2024-12-16

**Authors:** Hui Sun, Chenwei Wang, Jun Yang, Bin-Bin Zhang, Shaolin Xiong, Yi-Han Iris Yin, Yuan Liu, Ye Li, Wangchen Xue, Zhen-Yu Yan, Chen Zhang, Wenjun Tan, Haiwu Pan, Jiacong Liu, Huaqing Cheng, Yanqiu Zhang, Jingwei Hu, Chao Zheng, Zhenghua An, Ce Cai, Zhiming Cai, Lei Hu, Chichuan Jin, Dongyue Li, Xinqiao Li, Heyang Liu, Mingjun Liu, Wenxi Peng, Liming Song, Shengli Sun, Xiaojin Sun, Xilu Wang, Xiangyang Wen, Shuo Xiao, Shuxu Yi, Fan Zhang, Wenda Zhang, Xiaofeng Zhang, Yonghe Zhang, Donghua Zhao, Shijie Zheng, Zhixing Ling, Shuang-Nan Zhang, Weimin Yuan, Bing Zhang

**Affiliations:** National Astronomical Observatories, Chinese Academy of Sciences, Beijing 100101, China; Key Laboratory of Particle Astrophysics, Institute of High Energy Physics, Chinese Academy of Sciences, Beijing 100049, China; University of Chinese Academy of Sciences, Chinese Academy of Sciences, Beijing 100049, China; School of Astronomy and Space Science, Nanjing University, Nanjing 210023, China; Key Laboratory of Modern Astronomy and Astrophysics (Nanjing University), Ministry of Education, Nanjing 210022, China; School of Astronomy and Space Science, Nanjing University, Nanjing 210023, China; Key Laboratory of Modern Astronomy and Astrophysics (Nanjing University), Ministry of Education, Nanjing 210022, China; Purple Mountain Observatory, Chinese Academy of Sciences, Nanjing 210023, China; Key Laboratory of Particle Astrophysics, Institute of High Energy Physics, Chinese Academy of Sciences, Beijing 100049, China; School of Astronomy and Space Science, Nanjing University, Nanjing 210023, China; National Astronomical Observatories, Chinese Academy of Sciences, Beijing 100101, China; Purple Mountain Observatory, Chinese Academy of Sciences, Nanjing 210023, China; Key Laboratory of Particle Astrophysics, Institute of High Energy Physics, Chinese Academy of Sciences, Beijing 100049, China; University of Chinese Academy of Sciences, Chinese Academy of Sciences, Beijing 100049, China; School of Astronomy and Space Science, Nanjing University, Nanjing 210023, China; National Astronomical Observatories, Chinese Academy of Sciences, Beijing 100101, China; University of Chinese Academy of Sciences, Chinese Academy of Sciences, Beijing 100049, China; Key Laboratory of Particle Astrophysics, Institute of High Energy Physics, Chinese Academy of Sciences, Beijing 100049, China; University of Chinese Academy of Sciences, Chinese Academy of Sciences, Beijing 100049, China; National Astronomical Observatories, Chinese Academy of Sciences, Beijing 100101, China; Key Laboratory of Particle Astrophysics, Institute of High Energy Physics, Chinese Academy of Sciences, Beijing 100049, China; University of Chinese Academy of Sciences, Chinese Academy of Sciences, Beijing 100049, China; National Astronomical Observatories, Chinese Academy of Sciences, Beijing 100101, China; Key Laboratory of Particle Astrophysics, Institute of High Energy Physics, Chinese Academy of Sciences, Beijing 100049, China; University of Chinese Academy of Sciences, Chinese Academy of Sciences, Beijing 100049, China; National Astronomical Observatories, Chinese Academy of Sciences, Beijing 100101, China; Key Laboratory of Particle Astrophysics, Institute of High Energy Physics, Chinese Academy of Sciences, Beijing 100049, China; University of Chinese Academy of Sciences, Chinese Academy of Sciences, Beijing 100049, China; Key Laboratory of Particle Astrophysics, Institute of High Energy Physics, Chinese Academy of Sciences, Beijing 100049, China; College of Physics and Hebei Key Laboratory of Photophysics Research and Application, Hebei Normal University, Shijiazhuang 050024, China; Innovation Academy for Microsatellites, Chinese Academy of Sciences, Shanghai 201304, China; Purple Mountain Observatory, Chinese Academy of Sciences, Nanjing 210023, China; National Astronomical Observatories, Chinese Academy of Sciences, Beijing 100101, China; University of Chinese Academy of Sciences, Chinese Academy of Sciences, Beijing 100049, China; National Astronomical Observatories, Chinese Academy of Sciences, Beijing 100101, China; Key Laboratory of Particle Astrophysics, Institute of High Energy Physics, Chinese Academy of Sciences, Beijing 100049, China; National Astronomical Observatories, Chinese Academy of Sciences, Beijing 100101, China; National Astronomical Observatories, Chinese Academy of Sciences, Beijing 100101, China; University of Chinese Academy of Sciences, Chinese Academy of Sciences, Beijing 100049, China; Key Laboratory of Particle Astrophysics, Institute of High Energy Physics, Chinese Academy of Sciences, Beijing 100049, China; Key Laboratory of Particle Astrophysics, Institute of High Energy Physics, Chinese Academy of Sciences, Beijing 100049, China; University of Chinese Academy of Sciences, Chinese Academy of Sciences, Beijing 100049, China; Shanghai Institute of Technical Physics, Chinese Academy of Sciences, Shanghai 200083, China; Shanghai Institute of Technical Physics, Chinese Academy of Sciences, Shanghai 200083, China; Key Laboratory of Particle Astrophysics, Institute of High Energy Physics, Chinese Academy of Sciences, Beijing 100049, China; Key Laboratory of Particle Astrophysics, Institute of High Energy Physics, Chinese Academy of Sciences, Beijing 100049, China; Guizhou Provincial Key Laboratory of Radio Astronomy and Data Processing, Guizhou Normal University, Guiyang 550001, China; Key Laboratory of Particle Astrophysics, Institute of High Energy Physics, Chinese Academy of Sciences, Beijing 100049, China; Key Laboratory of Particle Astrophysics, Institute of High Energy Physics, Chinese Academy of Sciences, Beijing 100049, China; National Astronomical Observatories, Chinese Academy of Sciences, Beijing 100101, China; Innovation Academy for Microsatellites, Chinese Academy of Sciences, Shanghai 201304, China; Innovation Academy for Microsatellites, Chinese Academy of Sciences, Shanghai 201304, China; National Astronomical Observatories, Chinese Academy of Sciences, Beijing 100101, China; Key Laboratory of Particle Astrophysics, Institute of High Energy Physics, Chinese Academy of Sciences, Beijing 100049, China; National Astronomical Observatories, Chinese Academy of Sciences, Beijing 100101, China; University of Chinese Academy of Sciences, Chinese Academy of Sciences, Beijing 100049, China; Key Laboratory of Particle Astrophysics, Institute of High Energy Physics, Chinese Academy of Sciences, Beijing 100049, China; University of Chinese Academy of Sciences, Chinese Academy of Sciences, Beijing 100049, China; National Astronomical Observatories, Chinese Academy of Sciences, Beijing 100101, China; University of Chinese Academy of Sciences, Chinese Academy of Sciences, Beijing 100049, China; Nevada Center for Astrophysics, University of Nevada, Las Vegas, NV 89154, USA; Department of Physics and Astronomy, University of Nevada, Las Vegas, NV 89154, USA

**Keywords:** gamma-ray burst, magnetar, X-ray emission, compact binary merger

## Abstract

The central engine that powers gamma-ray bursts (GRBs), the most powerful explosions in the universe, is still not identified. Besides hyper-accreting black holes, rapidly spinning and highly magnetized neutron stars, known as millisecond magnetars, have been suggested to power both long and short GRBs. The presence of a magnetar engine following compact star mergers is of particular interest as it would provide essential constraints on the poorly understood equation of state for neutron stars. Indirect indications of a magnetar engine in these merger sources have been observed in the form of plateau features present in the X-ray afterglow light curves of some short GRBs. Additionally, some X-ray transients lacking gamma-ray bursts have been identified as potential magnetar candidates originating from compact star mergers. Nevertheless, smoking-gun evidence is still lacking for a magnetar engine in short GRBs, and associated theoretical challenges have been raised. Here we present a comprehensive analysis of the broad-band prompt emission data of the peculiar, very bright GRB 230307A. Despite its apparently long duration, the prompt emission and host galaxy properties are consistent with a compact star merger origin, as suggested by its association with a kilonova. Intriguingly, an extended X-ray emission component shows up as the $\gamma$-ray emission dies out, signifying the likely emergence of a magnetar central engine. We also identify an achromatic temporal break in the high-energy band during the prompt emission phase, which was never observed in previous bursts and reveals a narrow jet with a half opening angle of $\sim\! 3.4^\circ (R_{\rm GRB}/10^{15}{\rm cm})^{-1/2}$, where $R_{\rm GRB}$ is the GRB prompt emission radius.

## INTRODUCTION

**Figure 1.**
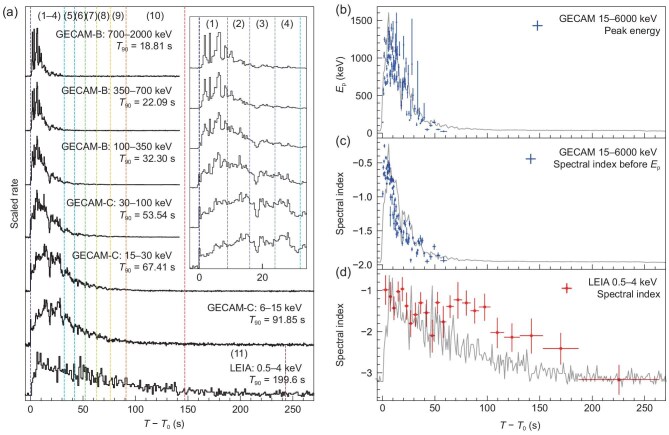
The temporal and spectral behaviors of GRB 230307A. (a) The multi-wavelength net light curves of GRB 230307A. The spectral energy distribution time intervals are demarcated by vertical dashed lines, accompanied by numerical labels at the top. Insets within the panel provide detailed profiles of prompt light curves in GECAM energy bands. (b) The evolution of the peak energy $E_{\rm p}$ and (c) the spectral power-law index derived from GECAM spectral fittings in 15–6000 keV. (d) The evolution of the spectral power-law index derived from LEIA spectral fittings in 0.5–4 keV. The light curves (gray lines) are plotted in the background as a reference. All error bars represent the 1$\sigma$ confidence level.

At 15:44:06.650 UT on 7 March 2023 (denoted $T_0$) the Gravitational Wave High-Energy Electromagnetic Counterpart All-Sky Monitor (GECAM) [1,2] was triggered by the extremely bright GRB 230307A [3] (Section S1). The burst was also reported by the *Fermi* Gamma-Ray Burst Monitor [4]. Utilizing the unsaturated GECAM data, we determined the burst’s duration ($T_{90}$) to be $41.52 \pm 0.03$ s in the 10–1000-keV energy range (Table 1; see Fig. 1a for the energy-band-dependent light curves). The peak flux and total fluence in the same energy range were found to be $4.26_{-0.07}^{+0.08} \times 10^{-4}\rm erg\,cm^{-2}s^{-1}$ and $(3.10 \pm 0.01) \times 10^{-3}\rm erg\,cm^{-2}$, respectively, making it the second brightest GRB observed, only dwarfed by the brightest-of-all-time GRB 221009A [5]. The pathfinder of the Einstein Probe mission [6], named the Lobster Eye Imager for Astronomy (LEIA) [7,8], with its large field of view of 340 $\rm deg^2$, caught the prompt emission of this burst in the soft X-ray band (0.5–4 keV) exactly at its trigger time [9], revealing a significantly longer duration of $199.6_{-2.2}^{+5.1}$ s and a peak flux of $3.6_{-0.5}^{+0.6} \times 10^{-7}\rm erg\,cm^{-2}s^{-1}$ (Fig. 1(a) and Table 1). Subsequent follow-up observations [10] indicate that the burst is most likely associated with a nearby galaxy at a redshift of $z=0.065$. Despite its long duration, the association of a kilonova signature [10,11] implies that this burst originates from a binary compact star merger.

**Table 1.** Observational properties. All errors represent the 1$\sigma$ uncertainties.

Observed properties	GRB 230307A
**Gamma ray (10–1000 keV)**	
Duration ($\rm s$)	$41.52 \pm 0.03$
Effective amplitude	$1.23 \pm 0.07$
Minimum variability timescale ($\rm ms$)	9.35
Rest-frame spectral lag$^{*}$ ($\rm ms$)	$1.6_{-1.2}^{+1.4}$
Spectral index $\alpha _1$	$-0.92_{-0.03}^{+0.05}$
Spectral index $\alpha _2$	$-1.274_{-0.008}^{+0.005}$
Spectral index $\beta$	$-3.85_{-0.09}^{+0.03}$
Break energy $E_{\rm b}$ ($\rm keV$)	$24_{-2}^{+3}$
Peak energy $E_{\rm p}$ ($\rm keV$)	$1052_{-8}^{+16}$
Peak flux ($\rm erg\,cm^{-2}s^{-1}$)	$4.26_{-0.07}^{+0.08} \times 10^{-4}$
Total fluence ($\rm erg\,cm^{-2}$)	$(3.10\pm 0.01) \times 10^{-3}$
Peak luminosity ($\rm erg\,s^{-1}$)	$4.64_{-0.08}^{+0.09} \times 10^{51}$
Isotropic energy ($\rm erg$)	$(3.18\pm 0.01) \times 10^{52}$
**Soft X-ray (0.5–4 keV)**	
Duration ($\rm s$)	$199.6_{-2.2}^{+5.1}$
Spectral index $\alpha$	$-1.70_{-0.06}^{+0.06}$
Peak flux ($\rm erg\,cm^{-2}s^{-1}$)	$3.6_{-0.5}^{+0.6} \times 10^{-7}$
Total fluence ($\rm erg\,cm^{-2}$)	$2.24_{-0.06}^{+0.07} \times 10^{-5}$
Peak luminosity ($\rm erg\,s^{-1}$)	$3.9_{-0.5}^{+0.6} \times 10^{48}$
Isotropic energy ($\rm erg$)	$2.44_{-0.06}^{+0.07} \times 10^{50}$
**Host galaxy**	
Redshift	0.065
Half-light radius ($\rm kpc$)	4.0
Offset ($\rm kpc$)	36.60
Normalized offset	9.2
Probability of chance coincidence	0.11
**Associations**	
Kilonova	Yes
Supernova	No

$^{*}$ The rest-frame spectral lag is measured between rest-frame energy bands 100–150 and 200–250 keV.

## RESULTS

### Compact star merger origin

The broad-band (0.5–6000 keV; see Section S2) prompt emission data we have collected from GECAM and LEIA also provide supportive evidence for a compact star merger origin. The burst’s placement on various correlation diagrams is consistent with so-called type-I GRBs [12], i.e. those with a compact star merger origin (see Section S5 and Fig. S3 within the online supplementary material). First, its relatively small minimum variability timescale is more consistent with type-I GRBs. Second, it deviates from the Amati relation of type-II GRBs (massive star core collapse origin), but firmly falls into the 1$\sigma$ scattering region of type-I GRBs (see also [11]). Third, it is a significant outlier of the anti-correlation between the spectral lags and peak luminosities of type-II GRBs, but is mixed in with other type-I GRBs. Besides, the optical host galaxy data [10] add yet one more support: the location of the burst has a significant offset from the host galaxy, which is at odds with type-II GRBs, but is fully consistent with type-I GRBs. This is the second strong instance for long-duration type-I GRBs after GRB 211211A [13–17].

### Gamma-ray properties

**Figure 2.**
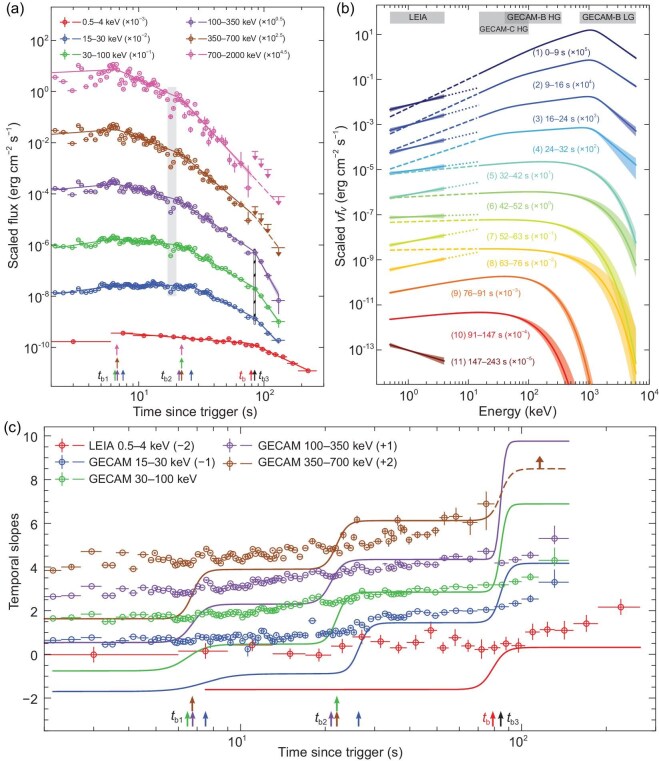
The flux light curves and spectral energy distributions (SEDs) of GRB 230307A. (a) The multi-wavelength flux light curves. The fluxes (data points) are derived from time-resolved spectral fittings, and the lines represent the best fits of smoothly broken power-law (SBPL) functions to the multi-wavelength flux light curves. The downward arrows represent the 3$\sigma$ upper limits of fluxes. The gray shaded area marks the dip phase (17–20 s since the trigger time). The black hatched area represents the achromatic break in GECAM energy bands. (b) The evolution of SEDs. The SEDs are derived from the spectral fittings at different time intervals, as indicated by the labels. The dotted and dashed lines represent the natural extrapolations of the best-fit models of LEIA and GECAM independent spectral fittings, respectively. (c) The temporal slopes. The data points ($\hat{\beta }+2$) represent the temporal slopes predicted by the curvature effect, and the lines ($\hat{\alpha }$) depict the temporal slopes corresponding to the SBPL fits to the multi-wavelength flux light curves. In (a) and (c), all the break times derived from the SBPL fits to the multi-wavelength flux light curves are labeled by the upward arrows. All error bars on data points represent their 1$\sigma$ confidence levels. All shaded areas around the best-fit lines represent their 1$\sigma$ confidence bands.

With the broad-band coverage jointly provided by GECAM-B/GECAM-C and LEIA throughout the prompt emission phase, one can perform a detailed temporal and spectral analysis of the data of GRB 230307A (Fig. 1 and see Section S4). The light curves in the energy range of GECAM-B and GECAM-C exhibit synchronized pulses with matching peak and dip features (Fig. 1(a)). The time-resolved spectrum in the 15–6000-keV band displays significant evolution (Fig. 1(b) and (c)), aligning with the ‘intensity tracking’ pattern (i.e. the peak energy tracks the intensity evolution [18]). When plotting the energy flux light curves in the logarithmic-logarithmic space (Fig. 2(a)), a break is identified around 21–26 s post-trigger in all five GECAM bands (15–30, 30–100, 100–350, 350–700 and 700–2000 keV), after which the light curves decay with indices ($\hat{\alpha }$) of $2.45_{-0.02}^{+0.02}$, $2.85_{-0.01}^{+0.01}$, $3.34_{-0.02}^{+0.02}$, $4.12_{-0.06}^{+0.06}$ and $5.3_{-0.3}^{+0.3}$ in the convention of $F_{\rm \nu } \propto t^{-\hat{\alpha }}\nu ^{-\hat{\beta }}$ for these bands, respectively (see Section S6 and Table 4 within the online supplementary material). These measured slopes are fully consistent with the theoretical prediction of the relationship between the temporal decay slope ($\hat{\alpha }$) and spectral slope ($\hat{\beta }$) solely due to the high-latitude emission after a sudden cessation of prompt emission, namely, $\hat{\alpha } = \hat{\beta } + 2$ (the so-called curvature effect [19]). This suggests that the prompt high-energy emission abruptly ceased or significantly reduced its emission amplitude at about 21–26 s post-trigger (see Section S7 and Fig. 2(c)). The variability observed in the light curves after the cessation of the central engine is naturally expected within the framework of the internal-collision-induced magnetic reconnection and turbulence model, because there are mini-jet emissions at high latitudes [20,21]. There is an additional achromatic break around 84 s, after which the three light curves (15–30, 30–100 and 100–350 keV) decay with much steeper slopes (Fig. 2(a) and (c)). This is consistent with the edge effect of a narrow jet that powers the prompt $\gamma$-ray emission, with a half opening angle of $\sim \! 3.4^\circ (R_{\rm GRB}/10^{15}{\rm cm})^{-1/2}$, where $R_{\rm GRB}$ is the unknown GRB prompt emission radius from the central engine (see Section S10). The jet opening angle is generally consistent with that derived from multi-band modeling of the afterglows [11], where the jet break is observed at around a few days. This brings the collimation-corrected jet energy to $\sim \! 5.6\times 10^{49}$ erg, typical for type-I GRBs [22].

### X-ray properties

In contrast to the hard X-rays and gamma rays, the soft X-ray emission in the 0.5–4-keV LEIA band exhibits a different behavior. The emission sustains for a much longer duration of $> \!250$ s in the form of a plateau followed by a decline. Its spectrum shows much less significant evolution within the first 100 s (see Fig. 1(d), and Table S1 within the online supplementary material) compared to the high-energy GECAM spectrum. Notably, its spectral shape from the beginning up to $\sim \!$76 s deviates strongly from the extrapolation to lower energies of the spectral energy distributions derived from the GECAM data (see Section S8 and Fig. 2(b)). These deviations cannot be easily ascribed to a simple spectral break at low energies as sometimes seen in GRBs [23], but rather hint at a different radiation process dominating the LEIA band. As the high-energy emission suddenly ceases at around 21–26 s (with the decay slope controlled by the curvature effect), the late decay slope in the LEIA band is shallower than the curvature effect prediction, suggesting an intrinsic temporal evolution from the central engine (Fig. 2(c)). These facts suggest that the LEIA-band soft X-ray emission comes from a distinct emission component from the GRB, which emerges already from the onset of the burst (Fig. 2(a)). After 76 s, as the $\gamma$-ray emission dies out, the joint LEIA and GECAM spectra can be well fitted using a single cutoff power-law model (Fig. 2(b)). This indicates that the entire spectra after 76 s is dominated by the new emergent component observed by LEIA.

### Magnetar emergence

**Figure 3.**
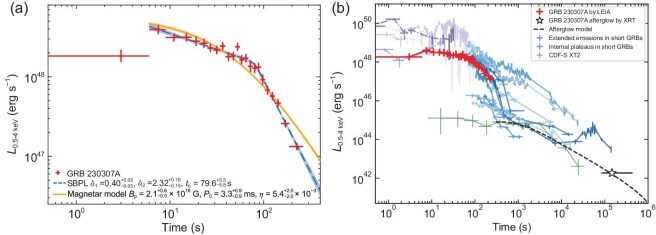
X-ray luminosity light curve of GRB 230307A. (a) The unabsorbed X-ray luminosity light curve in the energy range of 0.5–4 keV, excluding the first data point, is fitted using both the smoothly broken power-law model and the magnetar dipole radiation model, as described in Section S13. The shaded area represents the 1$\sigma$ confidence bands. (b) GRB 230307A compared with the X-ray afterglows of the internal plateau sample (solid line) and the extended emissions (dash-dotted line) in short GRBs [24] and CDF-S XT2, all corrected to the LEIA energy band. The Swift/XRT-detected X-ray afterglow of GRB 230307A is represented as an unfilled black star. The extrapolation of the afterglow model [11] is plotted as a black dashed curve, which is significantly below the LEIA data points, indicating that the LEIA emission may not be the early afterglow emission (see Section S12).

A smoothly broken power-law fit to the LEIA light curve gives decay slopes of $0.40_{-0.05}^{+0.05}$ and $2.32_{-0.15}^{+0.16}$ before and after the break time at $79.6_{-5.8}^{+5.5}$ s (see Section S13 and Fig. 3(a)). This pattern is generally consistent with the magnetic dipole spin-down law of a newborn, rapidly spinning magnetar. The best fit of the luminosity light curve with the magnetar model [24,25] yields a dipole magnetic field of $2.1^{+0.6}_{-0.5} \times 10^{16}$ G, an initial spin period of $3.3^{+0.9}_{-0.9}$ ms and a radiation efficiency of $5.4^{+2.0}_{-2.0}\times 10^{-3}$ (Fig. 3(a)). An X-ray plateau may also be interpreted within a black hole engine with a long-term accretion disk [26]. However, such a long-lived disk would eventually be quickly evaporated so that the jet engine would cease abruptly. The fact that the final decay slope of the LEIA light curve is shallower than the curvature effect prediction rules out such a possibility and reinforces the magnetar interpretation. The considerable discrepancy between the extrapolation of the afterglow model [11] and the observed data suggests that the soft X-ray component is unlikely to be the X-ray afterglow (Fig. 3(b)). Further modeling, by introducing a low-energy spectral break due to synchrotron self-absorption at low energies [15,27], indicates that the entire spectral energy distribution of the prompt emission can be interpreted by a combination of the prompt emission, which drops quickly at low energies, and a new magnetar component (see Section S9).

## DISCUSSION

Indirect evidence of a magnetar engine in compact star mergers has been collected before in the form of extended emissions or internal plateaus in short GRBs [28,24] and some short-GRB-less X-ray transients such as CDF-S XT2 [29]. Figure 3(b) shows the comparison of the X-ray luminosity light curves between GRB 230307A and other magnetar candidates. It shows that GRB 230307A is consistent with the other sources, but displays the full light curve right from the trigger directly in the X-ray band, thanks to the prompt detection by the wide-field X-ray camera of LEIA. It reveals the details of the emergence of the magnetar emission component and lends further support to the magnetar interpretation of other events. GRB 230307A marks the first simultaneous observation of a compact object merger in both X-ray and gamma-ray energies. In the near future, the synergy between GRB monitors and wide-field soft X-ray telescopes (such as the Einstein Probe) may detect more cases and will generally provide more observational information to diagnose the physics of GRBs during the prompt emission stage.

The identification of a magnetar engine from a merger event suggests that the neutron star (NS) equation of state is relatively stiff [30,31]. The association with a regular kilonova [10,11] suggests that the energy injection into the ejecta from the magnetar engine is moderate (Section S13). The magnetar engine also challenges modelers who currently fail to generate a relativistic jet from new-born magnetars [32]. One possibility is that a highly magnetized jet is launched seconds after the birth of the magnetar when the proto-neutron star cools down and the wind becomes clean enough [33]. In any case, the concrete progenitor of GRB 230307A remains an enigma. With a magnetar engine, the progenitor can only be a binary neutron star merger or a (near-Chandrasekhar-limit) white dwarf–NS merger [13]. For the former possibility, one must explain why this burst is particularly long. A ‘tip-of-the-iceberg’ test [34] suggests that it is hard to make this burst a bright short GRB when one arbitrarily raises the background flux or moves the source to higher redshift (see Section S3 and Table 1). For the latter scenario, the fact that the light curves and spectral evolution between GRBs 230307A and 211211A [13] do not fully resemble each other would suggest that the mechanism must be able to produce diverse light curves. Unfortunately, GRB 230307A was detected prior to the fourth operation run (O4) of the LIGO-Virgo-KAGRA network. Future multi-messenger observations of similar events hold the promise of eventually unveiling the identity of the progenitor of these peculiar systems [35].

## Supplementary Material

nwae401_Supplemental_File

## References

[1] Li XQ, Wen XY, An ZH et al. The technology for detection of gamma-ray burst with GECAM satellite. Radiat Detect Technol Methods 2021; 6: 12–25.

[2] Zhang D, Zheng C, Liu J et al. The performance of SiPM-based gamma-ray detector (GRD) of GECAM-C. Nucl Instrum Methods Phys Res 2023; 1056: 168586. 10.48550/arXiv.2303.00537

[3] Xiong S, Wang C, Huang Y et al. GRB 230307A: GECAM detection of an extremely bright burst. GRB Coordinates Network 2023; 33406.

[4] Fermi GBM Team . GRB 230307A: Fermi GBM final real-time localization. GRB Coordinates Network 2023; 33405.

[5] An ZH, Antier S, Bi XZ et al. Insight-HXMT and GECAM-C observations of the brightest-of-all-time GRB 221009A. arXiv: 2303.01203.

[6] Yuan W, Zhang C, Chen Y et al. The Einstein probe mission. In: Bambi C, Santangelo A (eds.). Handbook of X-Ray and Gamma-Ray Astrophysics. Singapore: Springer, 2024, 1171–200.

[7] Zhang C, Ling ZX, Sun XJ et al. First wide field-of-view X-ray observations by a lobster-eye focusing telescope in orbit. Astrophys J Let 2022; 941: L2. 10.3847/2041-8213/aca32f

[8] Ling ZX, Sun XJ, Zhang C et al. The Lobster Eye Imager for Astronomy Onboard the SATech-01 Satellite. Res Astron Astrophys 2023; 23: 095007. 10.1088/1674-4527/acd593

[9] Liu MJ, Wang YL, Liu Y et al. GRB 230307A: soft X-ray detection with LEIA. GRB Coordinates Network 2023; 33466.

[10] Levan AJ, Gompertz BP, Salafia OS et al. Heavy-element production in a compact object merger observed by JWST. Nature 2024; 626: 737–741. 10.48550/arXiv.2307.02098 37879361 PMC10881391

[11] Yang YH, Troja E, O’Connor B et al. A lanthanide-rich kilonova in the aftermath of a long gamma-ray burst. Nature 2024; 626: 742–5. 10.1038/s41586-023-06979-5 38383623

[12] Zhang B, Zhang BB, Virgili FJ et al. Discerning the physical origins of cosmological gamma-ray bursts based on multiple observational criteria: the cases of *z* $= 6.7$ GRB 080913, *z* $= 8.2$ GRB 090423, and some short/hard GRBs. Astrophys J 2009; 703: 1696–724. 10.1088/0004-637X/703/2/1696

[13] Yang J, Ai S, Zhang BB et al. A long-duration gamma-ray burst with a peculiar origin. Nature 2022; 612: 232–5. 10.1038/s41586-022-05403-8 36477130

[14] Rastinejad JC, Gompertz BP, Levan AJ et al. A kilonova following a long-duration gamma-ray burst at 350 Mpc. Nature 2022; 612: 223–7. 10.1038/s41586-022-05390-w 36477128

[15] Troja E, Fryer CL, O’Connor B et al. A nearby long gamma-ray burst from a merger of compact objects. Nature 2022; 612: 228–31. 10.1038/s41586-022-05327-3 36477127 PMC9729102

[16] Mei A, Banerjee B, Oganesyan G et al. Gigaelectronvolt emission from a compact binary merger. Nature 2022; 612: 236–9. 10.1038/s41586-022-05404-7 36477131

[17] Dichiara S, Tsang D, Troja E et al. A luminous precursor in the extremely bright GRB 230307A. Astrophys J Let 2023; 954: L29. 10.3847/2041-8213/acf21d

[18] Golenetskii SV, Mazets EP, Aptekar RL et al. Correlation between luminosity and temperature in $\gamma$-ray burst sources. Nature 1983; 306: 451–3. 10.1038/306451a0

[19] Kumar P, Panaitescu A. Afterglow Emission from naked gamma-ray bursts. Astrophys J Let 2000; 541: L51–4. 10.1086/312905

[20] Zhang B, Yan H. The internal-collision-induced magnetic reconnection and turbulence (ICMART) model of gamma-ray bursts. Astrophys J 2011; 726: 90. 10.1088/0004-637X/726/2/90

[21] Zhang B, Zhang B. Gamma-ray burst prompt emission light curves and power density spectra in the ICMART model. Astrophys J 2014; 782: 92. 10.1088/0004-637X/782/2/92

[22] Wang XG, Zhang B, Liang EW et al. Gamma-ray burst jet breaks revisited. Astrophys J 2018; 859: 160. 10.3847/1538-4357/aabc13

[23] Oganesyan G, Nava L, Ghirlanda G et al. Detection of low-energy breaks in gamma-ray burst prompt emission spectra. Astrophys J 2017; 846: 137. 10.3847/1538-4357/aa831e

[24] Lü HJ, Zhang B, Lei WH et al. The millisecond magnetar central engine in short GRBs. Astrophys J 2015; 805: 89. 10.1088/0004-637X/805/2/89

[25] Zhang B, Mészáros P. Gamma-ray burst afterglow with continuous energy injection: signature of a highly magnetized millisecond pulsar. Astrophys J Let 2001; 552: L35–8. 10.1086/320255

[26] Lu W, Quataert E. Late-time accretion in neutron star mergers: implications for short gamma-ray bursts and kilonovae. Mon Not R Astron Soc 2023; 522: 5848–61. 10.1093/mnras/stad1336

[27] Shen RF, Zhang B. Prompt optical emission and synchrotron self-absorption constraints on emission site of GRBs. Mon Not R Astron Soc 2009; 398: 1936–50. 10.1111/j.1365-2966.2009.15212.x

[28] Rowlinson A, O’Brien PT, Metzger BD et al. Signatures of magnetar central engines in short GRB light curves. Mon Not R Astron Soc 2013; 430: 1061–87. 10.1093/mnras/sts683

[29] Xue YQ, Zheng XC, Li Y et al. A magnetar-powered X-ray transient as the aftermath of a binary neutron-star merger. Nature 2019; 568: 198–201. 10.1038/s41586-019-1079-5 30971846

[30] Gao H, Zhang B, Lü HJ. Constraints on binary neutron star merger product from short GRB observations. Phys Rev D 2016; 93: 044065. 10.1103/PhysRevD.93.044065

[31] Margalit B and Metzger BD . The multi-messenger matrix: the future of neutron star merger constraints on the nuclear equation of state. Astrophys J Let 2019; 880: L15. 10.3847/2041-8213/ab2ae2

[32] Ciolfi R . Collimated outflows from long-lived binary neutron star merger remnants. Mon Not R Astron Soc 2020; 495: L66–70. 10.1093/mnrasl/slaa062

[33] Metzger BD, Giannios D, Thompson TA et al. The protomagnetar model for gamma-ray bursts. Mon Not R Astron Soc 2011; 413: 2031–56. 10.1111/j.1365-2966.2011.18280.x

[34] Lü HJ, Zhang B, Liang EW et al. The ‘amplitude’ parameter of gamma-ray bursts and its implications for GRB classification. Mon Not R Astron Soc 2014; 442: 1922–9. 10.1093/mnras/stu982

[35] Yin YHI, Zhang BB, Sun H et al. GRB 211211A-like events and how gravitational waves may tell their origins. Astrophys J Let 2023; 954: L17. 10.3847/2041-8213/acf04a

