# Supplementary material for: Magnetar emergence in a peculiar gamma-ray burst from a compact star merger
Source: Natl Sci Rev. 2024 Dec 16;12(3):nwae401. doi: 10.1093/nsr/nwae401 (PMC11887859; doi:10.1093/nsr/nwae401)
Supplement: nwae401_Supplemental_File [file nwae401_supplemental_file.pdf]

Supplementary Material

Magnetar emergence in a peculiar gamma-ray burst from a compact star merger

H. Sun[1,†], C.-W. Wang[2,3,†], J. Yang[4,5,†], B.-B. Zhang[4,5,6,*], S.-L. Xiong[2,*], Y.-H. I. Yin[4], Y. Liu[1], Y. Li[6], W.-C. Xue[2,3], Z.-Y. Yan[4], C. Zhang[1,3], W.-J. Tan[2,3], H.-W. Pan[1], J.-C. Liu[2,3], H.-Q. Cheng[1], Y.-Q. Zhang[2,3], J.-W. Hu[1], C. Zheng[2,3], Z.-H. An[2], C. Cai[7], Z.-M. Cai[8], L. Hu[6], C. Jin[1,3], D.-Y. Li[1], X.-Q. Li[2], H.-Y. Liu[1], M. Liu[1,3], W.-X. Peng[2], L.-M. Song[2,3], S.-L. Sun[9], X.-J. Sun[9], X.-L. Wang[2], X.-Y. Wen[2], S. Xiao[10], S.-X. Yi[2], F. Zhang[2], W.-D. Zhang[1], X.-F. Zhang[8], Y.-H. Zhang[8], D.-H. Zhao[1], S.-J. Zheng[2], Z.-X. Ling[1,3,*], S.-N. Zhang[2,3], W. Yuan[1,3], B. Zhang[11,12,*]

[1]National Astronomical Observatories, Chinese Academy of Sciences, Beijing 100101, China

[2]Key Laboratory of Particle Astrophysics, Institute of High Energy Physics, Chinese Academy of Sciences, Beijing 100049, China

[3]University of Chinese Academy of Sciences, Chinese Academy of Sciences, Beijing 100049, China

[4]School of Astronomy and Space Science, Nanjing University, Nanjing 210023, China

[5]Key Laboratory of Modern Astronomy and Astrophysics (Nanjing University), Ministry of Education, Nanjing 210022, China

[6]Purple Mountain Observatory, Chinese Academy of Sciences, Nanjing 210023, China

[7]College of Physics and Hebei Key Laboratory of Photophysics Research and Application, Hebei Normal University, Shijiazhuang, Hebei 050024, China

[8]Innovation Academy for Microsatellites, Chinese Academy of Sciences, Shanghai, 201304, China

[9]Shanghai Institute of Technical Physics, Chinese Academy of Sciences, Shanghai, 200083, China

[10]Guizhou Provincial Key Laboratory of Radio Astronomy and Data Processing, Guizhou Normal University, Guiyang 550001, China

[11]Nevada Center for Astrophysics, University of Nevada Las Vegas, NV 89154, USA

[12]Department of Physics and Astronomy, University of Nevada Las Vegas, NV 89154, USA

October 23, 2024

[*]**Corresponding authors.** Email: bbzhang@nju.edu.cn; xiongsl@ihep.ac.cn; lingzhixing@nao.cas.cn; bing.zhang@unlv.edu

[†]**Equally contributed to this work.**

1 Multi-mission observations of GRB 230307A

GRB 230307A triggered in real-time the Gravitational wave high-energy Electromagnetic Counterpart All-sky Monitor (GECAM) and the *Fermi* Gamma-ray Burst Monitor (GBM) almost simultaneously[1]. The extreme brightness of GRB 230307A was first reported by GECAM-B with its real-time alert data downlinked by the Beidou satellite navigation system[1], which was subsequently confirmed by *Fermi*/GBM[2, 3] and Konus-Wind[4]. Preliminary location of this burst measured by GECAM is consistent with that of GBM within error[1]. Refined localization in gamma-ray band was provided by the Inter-Planetary Network triangulation[5] and later improved by follow-up observations of the X-ray Telescope (XRT) aboard the Neil Gehrels Swift Observatory[6, 7]. The Lobster Eye Imager for Astronomy (LEIA) detected the prompt emission of this burst in the 0.5–4 keV soft X-ray band[8]. The burst was also followed up by the Gemini-South[9, 10] and the James Webb Space Telescope (JWST) at several epochs[11, 12, 13], unveiling a fading afterglow with possible signatures of kilonova in the optical and infrared bands and a candidate host galaxy at a redshift of $z = 0.065$[14].

[1]GECAM trigger time is 2023-03-07T15:44:06.650 UTC, which is 21 ms earlier than that of *Fermi*/GBM.

2 Data reduction

LEIA. The Lobster Eye Imager for Astronomy[15, 16], a pathfinder of the Einstein Probe mission of the Chinese Academy of Sciences, is a wide-field ($18.6^\circ \times 18.6^\circ$) X-ray focusing telescope built from novel technology of lobster eye micro-pore optics. The instrument operates in the 0.5–4 keV soft X-ray band, with an energy resolution of 130 eV (at 1.25 keV) and a time resolution of 50 ms. LEIA is onboard the Space Advanced Technology demonstration satellite (SATech-01), which was launched on 2022 July 27 and is operating in a Sun-synchronous orbit with an altitude of 500 km and an inclination of 97.4°. Since LEIA operates only in the Earth's shadow to eliminate the effects of the Sun, the observable time out of the radiation belt at high geo-latitude regions is $\sim$1000 s for each orbit. The observation of GRB 230307A was conducted from 15:42:32 UT (94 s earlier than the GECAM trigger time) to 16:00:50 UT on 7 March 2023 with a net exposure of 761 s. GRB 230307A was detected within the extended field of view (about 0.6° outside the nominal field of view) of LEIA[8] (Supplementary Data Fig. 1).

The X-ray photon events were processed and calibrated using the data reduction software and calibration database (CALDB) designed for the Einstein Probe mission (Liu et al. in prep.). The CALDB is generated based on the results of on-ground and in-orbit calibration campaigns (Ref.[17] and Cheng et al. in prep.). The energy of each event was corrected using the bias and gain stored in CALDB. Bad/flaring pixels were also flagged. Single-, double-, triple-, and quadruple-events without anomalous flags were selected to form the cleaned event file. The image in the 0.5–4 keV range was extracted from the cleaned events (Supplementary Data Fig. 1a). The position of each photon was projected into celestial coordinates (J2000). The light curve and the spectrum of the source and background in a given time interval were extracted from the regions indicated in Supplementary Data Fig. 1a. Since the peak count rate is only 2 ct/frame over the extended source region, the pile-up effect is negligible in the LEIA data.

LEIA is the very first of its kind with a considerably large field of view ever flown. To fully characterize its instrumental performance before launch, a series of tests and calibrations have been performed for the mirror assembly and the detectors separately, and for the complete module of the LEIA instrument[15, 16, 18, 17]. The in-flight calibration, which took over 8 months, was performed after launch and aimed to achieve accurate source localization and flux derivation (Cheng et al. in prep). The systematic uncertainty of source positioning is $1-2$ arcmin and that of the effective area (of the central focal spot) is about 10 percent, both at the 90 percent confidence level. However, as LEIA detected the cruciform arms of the source image (point spread function, PSF) of GRB 230307A instead of its focal spot, in order to verify the effective area (of the photon extraction region on the arm, i.e. the red-dotted box in Supplementary Data Fig. 1a) employed in the spectral fitting, which is derived from simulations, we performed observations of the Crab nebula (the standard calibration source) for 5 orbits on March 14, 2023 with a total exposure time of 4398 seconds. The Crab nebula was placed at the same direction of GRB 230307A relative to the same detector in these observations,

and the same analysis procedure was conducted to the data. The fitted photon index and estimated flux of the Crab are consistent with those derived from previous in-flight calibrations (also using the Crab nebular) at a level of less than 10 percent. This value is consistent with the aforementioned systematic error.

GECAM. GECAM is a dedicated all-sky gamma-ray monitor constellation funded by the Chinese Academy of Sciences. The original GECAM mission[19] is composed of two microsatellites (GECAM-A and GECAM-B) launched in December 2020. GECAM-C[20] is the 3rd GECAM spacecraft, also onboard the SATech-01 satellite as LEIA. Each GECAM spacecraft has an all-sky field of view unblocked by the Earth, capable of triggering bursts in real-time[21] and distributing trigger alerts promptly with the Global Short Message Communication of Beidou satellite navigation system[22]. As the main instrument of GECAM, most gamma-ray detectors (GRDs) operate in two readout channels: high gain (HG) and low gain (LG), which are independent in terms of data processing, transmission, and dead-time[23]. Comprehensive ground and cross calibrations have been conducted on the GRDs of both GECAM-B and GECAM-C[24, 20, 25, 26]. Both GECAM-B and GECAM-C have charged particle detectors (CPDs) to monitor the space environment, which is designed to be sensitive to charged particles and much less sensitive to photons[27]. However, for some extremely bright GRBs, CPDs can record some photons.

GRB 230307A was detected by GECAM-B and GECAM-C, while GECAM-A was offline. GECAM-B was triggered by this burst and automatically distributed a trigger alert to General Coordinates Network (GCN) Notice about 1 minute post-trigger[2]. GECAM-C also made the real-time trigger onboard, while the trigger alert was disabled due to the high latitude region setting[20]. With the automatic pipeline processing the GECAM-B real-time alert data[28], we promptly noticed and reported that this burst features an extreme brightness[1], which initiated the follow-up observations.

Both GECAM-B and GECAM-C were working in inertial pointing mode during the course of GRB 230307A. Among all 25 GRDs of GECAM-B, GRD04 maintains a constant minimum incident angle of 12.7° throughout the duration of the burst. GRD01, GRD05 also exhibit small incident angles of 32.4°, 32.7°, respectively. Thus these three detectors are selected for subsequent analysis. For GECAM-B GRDs, the HG channel operates from $\sim$ 40 to 350 keV, while the LG channel from $\sim$ 700 keV to 6 MeV. Among all 12 GRDs of GECAM-C, GRD01 exhibits the most optimal incident angle of 10.1° throughout the burst and is selected in the subsequent analysis. For GECAM-C/GRD01, the HG channel operates from $\sim$ 6 to 350 keV. Since the detector response for 6–15 keV is affected by the electronics and is subject to further verification[26], we only use >15 keV for spectral analysis in this work.

During the detection of GRB 230307A, the background of GECAM-B was found to be very stable and could be well fitted by a first-order polynomial. However, GECAM-C experienced background evolution due to the motion of the satellite in orbit, which can also be seen from the light

[2]GECAM real-time alert for GRB 230307A: `https://gcn.gsfc.nasa.gov/other/160.gecam`

curve of CPDs. The background estimation methodology for GECAM-C/GRD01 involves fitting an empirical function with a combination of first and second-order exponential polynomials (i.e., $\exp(c_0 + c_1 t)+\exp(c_2 + c_3 t + c_4 t^2)$) (Supplementary Data Fig. 2a). Though the CPD is much less sensitive to photons, a clear profile of GRB 230307A can be seen on the residual of CPD background fitting owing to the extreme photon flux during the peak region of this GRB. The reasonable residuals at other time intervals rather than the peak time period of GRB 230307A indicate that the background of GECAM-C is well modelled and subtracted. More directly, this background estimation of GECAM-C is verified by the comparison with GECAM-B data (Supplementary Data Fig. 2b).

We note that GRB 230307A was so bright that the *Fermi*/GBM observation suffered from data saturation[29]. GECAM has dedicated designs to minimize data saturation for bright bursts[19, 30]. For GECAM, the engineering count rate records the number of events processed onboard, while the event count rate records the number of events received on ground. In the case of data saturation, these two count rates would differ significantly. As shown in Supplementary Data Fig. 2c, the negligible discrepancy between these two count rates, due to the limited digital accuracy on the count numbering, confirms no count loss in event data and indicates the absence of saturation for both GECAM-B and GECAM-C.

3 Temporal analysis

Duration. The light curves are obtained through the process of photon counts binning (Fig. 1). The light curve for LEIA is obtained in 0.5–4 keV with a bin size of 1 s. The light curves for GECAM-B are generated using the bin size of 0.4 s in the energy ranges of 100–350 keV, 350–700 keV, and 700–2000 keV, by combining the data from three selected detectors (namely, GRD04, GRD01, and GRD05). The GECAM-C light curves are derived by binning the photon counts from GRD01 with a bin size of 0.4 s in the energy ranges of 6–15 keV, 15–30 keV, and 30–100 keV. The burst duration, denoted as T_{90}, is determined by calculating the time interval between the epochs when the total accumulated net photon counts reach the 5% and 95% levels. The durations obtained from the multi-wavelength light curve are annotated in Fig. 1. It is found that the duration of the burst significantly increases towards the lower energy range. We also calculate the duration within 10–1000 keV based on the data from GECAM-B and list it in Table 1.

Amplitude parameter. The amplitude parameter[31] is a metric used to classify GRBs and is defined as $f = F_{\rm p}/F_{\rm b}$, denoting the ratio between the peak flux $F_{\rm p}$ and the background flux $F_{\rm b}$ at the same time epoch. A long-duration type II GRB may be disguised as a short-duration type I GRB due to the tip-of-iceberg effect. To distinguish intrinsically short-duration type I GRBs from ostensible short-duration type II GRBs, an effective amplitude parameter, $f_{\rm eff} = \epsilon f = F'_{\rm p}/F_{\rm b}$, can be defined for long-duration type II GRBs by quantifying the tip-of-iceberg effect, where $F'_{\rm p}$ is the

peak flux of a pseudo GRB whose amplitude is lower by a factor ϵ from an original long-duration type II GRB so that its duration is just shorter than 2 s. Generally speaking, the $f_{\rm eff}$ values of long-duration type II GRBs are systematically smaller than the f values of short-duration type I GRBs, thereby facilitating their distinction from one another. Utilizing the procedure presented in Ref.[31], the effective amplitude of GRB 230307A is determined to be $f_{\rm eff} = 1.23 \pm 0.07$ within the energy range of 10–1000 keV (Table 1). Such small $f_{\rm eff}$ value aligns with the characteristics typically exhibited by long-duration GRBs.

Variability. The minimum variability timescale (MVT) is defined as the shortest timescale of significant variation that exceeds statistical noise in the GRB temporal profile[32, 33]. It serves as an indicator of both the central engine's activity characteristics[34] and the geometric dimensions of the emitting region. The median values of the minimum variability timescale in the rest frame (i.e., $\mathrm{MVT}/(1+z)$) for type I and type II GRBs are found to be 10 ms and 45 ms, respectively. To determine the MVT, we utilize the Bayesian block algorithm[35] on the entire light curve within the 10–1000 keV energy range to identify the shortest block that satisfies the criterion of encompassing the rising phase of a pulse. We find that the MVT of GRB 230307A is about 9.35 ms (Table 1), which is more consistent with type I GRBs rather than type II GRBs in the distribution of the MVTs[32] (Supplementary Data Fig. 3a). We also utilize the continuous wavelet transform method to derive MVT and obtain a consistent outcome in accordance with the Bayesian block algorithm.

Spectral lag. Spectral lag refers to the time delay between the soft-band and hard-band background-subtracted light curves. It may be attributed to the curvature effect in the relativistic outflow. Upon reaching the observer, on-axis photons are boosted to higher energies, while off-axis photons receive a smaller boost and must travel a longer distance. Type II GRBs usually exhibit considerable spectral lags, while type I GRBs tend to have tiny lags[36, 37], indicating the difference in their emission region sizes. Measurement of the spectral lag can be achieved by determining the time delay corresponding to the maximum value of the cross-correlation function[38, 39, 40]. Following the treatment in Ref.[39], we use the background-subtracted light curves of GRB 230307A to measure the rest-frame lag between rest-frame energy bands 100–150 and 200–250 keV to be $1.6^{+1.4}_{-1.2}$ ms (Table 1). We note that GRB 230307A manifests a very tiny spectral lag, an indicator that points towards being a type I GRB (Supplementary Data Fig. 3c).

4 Spectral analysis

LEIA spectral fitting. We perform a detailed spectral analysis using the LEIA data. The energy channels in the range of 0.5–4 keV are utilized and re-binned to ensure that each energy bin contains at least ten counts. We employ three kinds of time segmentation approaches to extract LEIA spectra:

- LEIA S-I: the time interval from 0 to 250 s is divided into three slices (0–50 s, 50–150 s,

150–250 s) to investigate the possible evolution of X-ray absorption;

- LEIA S-II: 23 time slices with sufficient time resolution are obtained by accumulating 100 photon counts for each individual slice;
- LEIA S-III: 12 time slices with sufficient level of confidence are obtained by accumulating 200 photon counts for each individual slice. This partitioning is designed to align with the temporal divisions of the GECAM S-III spectra, enabling subsequent comparisons and joint spectral fitting.

For each of the above time slices, we generate the source spectrum and background spectrum, and the corresponding detector redistribution matrix and the ancillary response.

First, the spectra of LEIA S-I are individually fitted with the *XSPEC*[41] using the *tbabs*ztbabs*powerlaw* model, where the first and second components are responsible for the Galactic absorption and intrinsic absorption ($N_{\rm H}$), and the third one is a power law function in the observer's frame. We incorporate the absorption model[42] for the cross sections and abundances. We employ CSTAT[43] as the statistical metric to evaluate the likelihood of LEIA spectral fitting where both the source and background spectra are Poisson-distributed data. The column density of the Galactic absorption in the direction of the burst is fixed at 1.26×10^{21} cm^{-2}[44] and the redshift is fixed at 0.065. It is found that all three spectra of S-I can be reproduced reasonably well by the absorption modified power law model (Supplementary Data Fig. 1b). The best-fit values and confidence contours of the column density and photon index are shown in Supplementary Data Fig. 1c. The fitted column densities are generally consistent within their uncertainties, indicating no significant variations of the absorption feature within the observation of LEIA. We perform a simultaneous fitting of all S-III spectra to make full use of all spectra with sufficient level of confidence. A time-averaged absorption of $N_{\rm H} = 3.92 \times 10^{21}$ cm^{-2}, is obtained and thus fixed in all subsequent spectral analysis. This value is larger than that derived from the X-ray afterglow[45] at ~ 4.5 days. The early soft X-ray detection of this bright burst provides an opportunity to observe the absorption variation. Such variation in the absorption density has only been reported for a few GRBs[46]. Given that the burst is located at large galactocentric distances, the decrease in $N_{\rm H}$ over time may be associated with the dynamically evolving circumburst medium during the early afterglow phase.

We then proceed with the fitting of the LEIA S-II spectra. The obtained results for the photon index ($\Gamma_{\rm ph}$), normalization, and the corresponding fitting statistic are presented in Supplementary Data Table 1. By employing a redshift of $z = 0.065$, we further calculated the unabsorbed flux (Fig. 2a) and determined the luminosity for each S-II spectrum (Fig. 3a and 3b). Additionally, we cross-validated our findings by analyzing spectra with higher photon statistics (S-III), and found that the alternate spectra yielded consistent results in our analysis. We also perform a spectral fit to the time-averaged spectrum during the whole observation interval of S-II, and the derived power-law index ($\alpha = -\Gamma_{\rm ph}$) is shown in Table 1. Finally, the LEIA S-III spectra are employed for spectral energy distribution analysis.

GECAM spectral fitting. We conduct a thorough time-resolved and time-integrated spectral analysis using the data from the GRD04, GRD01 and GRD05 of GECAM-B, as well as the GRD01 of GECAM-C. Each GRD detector has two independent readout channels, namely, high gain (HG) and low gain (LG). We utilize both the high and low gain data of the GECAM-B detectors with effective energy ranges of 40–350 and 700–6000 keV, respectively. Additionally, we only use the high gain data of the GECAM-C detector with an effective energy range of 15–100 keV but ignored the channels within 35–42 keV around the Iodine K-edge at 38.9 keV. We employ three distinct time segmentation methods to extract GECAM spectra:

- GECAM S-I: the time interval of 1.6–43.2 s, in which 5% and 95% of the total net photons accumulate, is utilized as a single time slice for conducting time-integrated spectral analysis;
- GECAM S-II: the time interval of 0–147 s is divided into 99 time slices with sufficient photon counts and approximately equal signal-to-noise levels to study the spectral evolution;
- GECAM S-III: the time interval of 0–147 s is partitioned to match the time division of LEIA S-III for studying the spectral energy distributions. However, to ensure that there is at least five net photons per detector channel on average, we merge two bins, namely 91–113 s and 113–147 s, into a single bin. Consequently, the time interval of 0–147 s is divided into ten time slices.

For each of the above time slices, we generate a source spectrum, a background spectrum, and a response matrix for each gain mode of each detector. Then we perform spectral fitting by utilizing the Python package, *MySpecFit*, in accordance with the methodology outlined in Refs.[47, 48]. The *MySpecFit* package facilitates Bayesian parameter estimation by wrapping the widely-used Fortran nested sampling implementation *Multinest*[49, 50, 51, 52]. PGSTAT[41] is utilized for GECAM spectral fitting, which is appropriate for Poisson data in the source spectrum with Gaussian background in the background spectrum. To fit the observed spectra, seven empirical models ranging from simple to complex are adopted. Here, we establish the following conventions for the model parameters: $N(E)$ is the photon spectrum, A is the normalization parameter in units of photons cm^{-2} s^{-1} keV^{-1}, $E_{\rm p}$ is defined as the peak energy of νf_ν spectrum, β is the high-energy photon index above the peak energy, $E_{\rm b}$ and $E_{\rm c}$ are defined as the low-energy break and high-energy cutoff of photon spectrum respectively, α_1 and α_2 represent the spectral indices of the two low-energy power law segments smoothly connected at the break energy, and α represents the spectral index at low energies if the break energy does not exist. Then all the spectral models are listed as follows:

- The power law (PL):

$$N(E) = A\Big(\frac{E}{100\,\mathrm{keV}}\Big)^{\alpha} \tag{1}$$

- The cutoff power law (CPL):

$$N(E) = A\Big(\frac{E}{100\,\mathrm{keV}}\Big)^{\alpha}\exp\Big(-\frac{E}{E_{\rm c}}\Big), \tag{2}$$

where the peak energy E_p is related to the cutoff energy E_c through $E_\mathrm{p} = (2+\alpha)E_\mathrm{c}$.

- The Band function (BAND[53]):

$$N(E) = \begin{cases} A(\frac{E}{100\,\mathrm{keV}})^{\alpha}\exp(-\frac{E}{E_\mathrm{c}}),\ E < (\alpha-\beta)E_\mathrm{c}, \\ A\big[\frac{(\alpha-\beta)E_\mathrm{c}}{100\,\mathrm{keV}}\big]^{\alpha-\beta}\exp(\beta-\alpha)(\frac{E}{100\,\mathrm{keV}})^{\beta}, E \geq (\alpha-\beta)E_\mathrm{c}, \end{cases} \tag{3}$$

where the peak energy E_p is related to the cutoff energy E_c through $E_\mathrm{p} = (2+\alpha)E_\mathrm{c}$.

- The smoothly broken power law (SBPL[54]):

$$N(E) = AE_\mathrm{j}^{\alpha}\Big[\Big(\frac{E}{E_\mathrm{j}}\Big)^{-\alpha n} + \Big(\frac{E}{E_\mathrm{j}}\Big)^{-\beta n}\Big]^{-\frac{1}{n}}, \tag{4}$$

where $E_\mathrm{j} = E_\mathrm{p}(-\frac{\alpha+2}{\beta+2})^{\frac{1}{(\beta-\alpha)n}}$, and the smoothness parameter is fixed to $n = 2.69$.

- The Band function with high-energy exponential cutoff (CBAND[55]):

$$N(E) = \begin{cases} AE^{\alpha_1}\exp(-\frac{E}{E_1}),\ E \leq E_\mathrm{b}, \\ AE_\mathrm{b}^{\alpha_1-\alpha_2}\exp(\alpha_2-\alpha_1)E^{\alpha_2}\exp(-\frac{E}{E_2}),\ E > E_\mathrm{b}, \end{cases} \tag{5}$$

where the break energy is expressed as $E_\mathrm{b} = \frac{E_1E_2}{E_2-E_1}(\alpha_1-\alpha_2)$, and the peak energy is defined as $E_\mathrm{p} = (2+\alpha_2)E_2$.

- The smoothly broken power law with high-energy exponential cutoff (CSBPL):

$$N(E) = AE_\mathrm{b}^{\alpha_1}\Big[\Big(\frac{E}{E_\mathrm{b}}\Big)^{-\alpha_1 n} + \Big(\frac{E}{E_\mathrm{b}}\Big)^{-\alpha_2 n}\Big]^{-\frac{1}{n}}\exp\Big(-\frac{E}{E_\mathrm{c}}\Big), \tag{6}$$

where the peak energy E_p is related to the cutoff energy E_c through $E_\mathrm{p} = (2+\alpha_2)E_\mathrm{c}$, and the smoothness parameter is fixed to $n = 5.38$.

- The double smoothly broken power law (2SBPL[54]):

$$N(E) = AE_\mathrm{b}^{\alpha_1}\Big[\Big[\Big(\frac{E}{E_\mathrm{b}}\Big)^{-\alpha_1 n_1} + \Big(\frac{E}{E_\mathrm{b}}\Big)^{-\alpha_2 n_1}\Big]^{\frac{n_2}{n_1}} + \Big(\frac{E}{E_\mathrm{j}}\Big)^{-\beta n_2}\Big[\Big(\frac{E_\mathrm{j}}{E_\mathrm{b}}\Big)^{-\alpha_1 n_1} + \Big(\frac{E_\mathrm{j}}{E_\mathrm{b}}\Big)^{-\alpha_2 n_1}\Big]^{\frac{n_2}{n_1}}\Big]^{-\frac{1}{n_2}}, \tag{7}$$

where $E_\mathrm{j} = E_\mathrm{p}(-\frac{\alpha_2+2}{\beta+2})^{\frac{1}{(\beta-\alpha_2)n_2}}$, and the smoothness parameters are fixed to $n_1 = 5.38$ and $n_2 = 2.69$.

We then fit them to the spectral data in each time slice. We perform a model comparison based on the Bayesian Information Criterion[56] (BIC) with the following procedure:

1. A BIC value is calculated for each of the above models;
2. The minimal value, $\mathrm{BIC}_\mathrm{min}$, of the above seven BIC values is obtained;
3. Within the range of [$\mathrm{BIC}_\mathrm{min}$, $\mathrm{BIC}_\mathrm{min} + 2$], we check how many BIC values fall in that range.

4. If only one model's BIC falls in the range [$\mathrm{BIC_{min}}$, $\mathrm{BIC_{min}} + 2$], we select such a model as the best model.

5. If two or more models' BIC values fall in the range [$\mathrm{BIC_{min}}$, $\mathrm{BIC_{min}} + 2$], we consider those models can equally explain the data[57, 58, 59]. In this case, if those models include the CPL model, we simply select it as the best-fit model; otherwise, we select the one corresponding to $\mathrm{BIC_{min}}$.

Priority is given to CPL in step 5, as it allows us to study the spectral properties in the most consistent manner possible. Additionally, this consideration takes into account the fact that most GRBs' fine time-resolved spectra can indeed be described by a CPL model.

Supplementary Data Table 2 and 3 list the spectral fitting results of the best model and corresponding fitting statistics for GECAM spectra. It should be noted that we use cutoff energy as a substitute for peak energy when the 1σ lower limit of α_2 (or α) nears or falls below -2. The GECAM S-I spectrum can be best fitted by the 2SBPL model with spectral indices $\alpha_1 = -0.92^{+0.05}_{-0.03}$, $\alpha_2 = -1.274^{+0.005}_{-0.008}$, and $\beta = -3.85^{+0.03}_{-0.09}$, as well as the break energy $E_{\rm b} = 24^{+3}_{-2}$ keV and peak energy $E_{\rm p} = 1052^{+16}_{-8}$ keV. Fig. 1b and 1c illustrate the significant "intensity tracking" spectral evolution in terms of $E_{\rm p}$ and α_2 (or α) derived from GECAM S-II spectra, respectively. During the time interval before approximately 30 s, we notice that many time-resolved GECAM spectra necessitated the use of a model incorporating a low-energy break (i.e., CBAND, CSBPL and 2SBPL) for fitting. The break energy $E_{\rm b}$ exhibits a decreasing trend from approximately 200 to 30 keV, with the spectral indices (i.e., α_1 and α_2) on either side of the break energy distributed in the 1σ interval of [-0.9, -0.4] and [-1.6, -1.2], respectively. It should be noted that the fitting statistics are acceptable for all time-resolved GECAM S-II spectra, whereas for the time-integrated GECAM S-I spectrum and some GECAM S-III spectra, the relatively large statistics should not be over-interpreted but can be attributed to spectral evolution[60].

5 Classification

Variability timescale versus duration. The temporal variability timescale of GRBs may conceal imprints of central engine activity and energy dissipation processes. On average, the minimum variability timescale of short-duration type I GRBs is significantly shorter than that of long-duration type II GRBs, providing a new clue to distinguish the nature of GRB progenitors and central engines[32, 33]. We collected previous research samples, redrawn the MVT $- T_{90}$ diagram, and overlaid GRB 230307A and GRB 211211A on it (Supplementary Data Fig. 3a). It is noteworthy that these two GRBs are outliers as they fall outside the 3σ confidence contours of both the type I and type II populations. While in terms of the MVT distribution, we find that their MVTs are more consistent with that of type I GRBs.

Peak energy versus isotropic energy. The $E_{\rm p,z} - E_{\gamma,\rm iso}$ diagram serves as a unique classification scheme in the study of GRB energy characteristics, as different physical origins of GRBs typically follow distinct tracks[61, 62, 63]. We first replot the $E_{\rm p,z} - E_{\gamma,\rm iso}$ diagram (Supplementary Data Fig. 3b) based on previous samples of type I and type II GRBs with known redshifts[63]. Here, $E_{\rm p,z} = E_{\rm p}(1+z)$ represents the rest-frame peak energy, while $E_{\gamma,\rm iso}$ denotes the isotropic energy. The relations between $E_{\rm p,z}$ and $E_{\gamma,\rm iso}$ can be modeled using a linear relationship, $\log E_{\rm p,z} = b + k \log E_{\gamma,\rm iso}$, for both GRB samples. The fitting process is implemented using the Python module emcee[64], and the likelihood is determined using the orthogonal-distance-regression (ODR) method[65]. The ODR method is appropriate in this case as the data satisfy two criteria: (1) a Gaussian intrinsic scatter $\sigma_{\rm int}$ along the perpendicular direction; (2) independent errors σ_{x_i} and σ_{y_i} on both the x and y axes. The log-likelihood function can be expressed as

$$\ln\mathcal{L} = -\frac{1}{2}\sum_i \left[\ln(2\pi\sigma_i^2) + \frac{\Delta_i^2}{\sigma_i^2}\right], \tag{8}$$

with the perpendicular distance

$$\Delta_i^2 = \frac{(y_i - kx_i - b)^2}{k^2+1}, \tag{9}$$

and the total perpendicular uncertainties

$$\sigma_i^2 = \frac{k^2\sigma_{x_i}^2 + \sigma_{y_i}^2}{k^2+1} + \sigma_{\rm int}^2, \tag{10}$$

where the subscript i runs over all data points. The best-fitting parameters with 1σ uncertainties are $k_{\rm I} = 0.36^{+0.04}_{-0.05}$, $b_{\rm I} = -15.61^{+2.51}_{-2.14}$ and $\log\sigma_{\rm int,I} = -1.29^{+0.13}_{-0.13}$ for type I GRBs, and $k_{\rm II} = 0.39^{+0.02}_{-0.02}$, $b_{\rm II} = -17.82^{+0.93}_{-0.98}$ and $\log\sigma_{\rm int,II} = -1.42^{+0.05}_{-0.05}$ for type II GRBs. The best-fit correlations and corresponding 1σ intrinsic scattering regions are presented in Supplementary Data Fig. 3b. The peak energy of GRB 230307A is constrained to be $E_{\rm p} = 1052^{+16}_{-8}$ keV by the spectral fitting to GECAM S-I. Given a redshift of 0.065, the isotropic energy of GRB 230307A can be calculated as $E_{\gamma,\rm iso} = (3.18 \pm 0.01) \times 10^{52}$ erg based on the spectral fits to GECAM S-II. We overplot both long-duration GRB 230307A and GRB 211211A on the $E_{\rm p,z} - E_{\gamma,\rm iso}$ diagram. As can be seen from Supplementary Data Fig. 3b, GRB 211211A resides in an intermediate area between the tracks of type I and type II GRBs. The perpendicular distance, Δ, is approximately $1.5\sigma_{\rm int,I}$ away from the type I track and $1.6\sigma_{\rm int,II}$ away from the type II track. On the other hand, GRB 230307A is firmly located within the 1σ intrinsic scattering region of the type I GRB track. Specifically, the perpendicular distance is approximately $0.8\sigma_{\rm int,I}$ away from the type I track and $2.3\sigma_{\rm int,II}$ away from the type II track. In addition, GRB 230307A poses higher total energy and harder spectra, likely indicating a more intense merger event. We also consider the scenario where the host galaxy has a redshift of 3.87[13] and notice that in this case, the isotropic energy of GRB 230307A is $\sim 10^{56}$ erg, which is an order of magnitude larger than that of the brightest-of-all-time GRB 221009A[48, 66]. Such extremely high energy is very unlikely consistent with the known sample of GRBs.

Peak luminosity versus spectral lag. An anti-correlation exists between the spectral lag and peak luminosity in the sample of type II GRB with a positive spectral lag[38, 39]. Such anti-

correlation can serve as a physically ambiguous indicator, suggesting that GRBs with short spectral lags have higher peak luminosities. In general, type I GRBs deviate from the anti-correlation of type II GRBs, with type I GRBs tending to exhibit smaller spectral lags than type II GRBs at the same peak luminosity. The significant differences between the two in this regard make the peak luminosity versus spectral lag correlation a useful classification scheme. On the basis of the previous samples of type I and type II GRBs with known redshift[67, 39, 68], we replot the $L_{\gamma,\mathrm{iso}} - \tau_{\mathrm{z}}$ diagram, where $L_{\gamma,\mathrm{iso}}$ is isotropic peak luminosity and $\tau_{\mathrm{z}} = \tau/(1+z)$ is the rest-frame spectral lag (Supplementary Data Fig. 3c). The anti-correlation between $L_{\gamma,\mathrm{iso}}$ and τ_{z} for type II GRBs can be fit with the linear model $\log L_{\gamma,\mathrm{iso}} = b + k\log\tau_{\mathrm{z}}$. The ODR method[65] gives the best-fitting parameters with 1σ uncertainties to be $k = -0.94^{+0.07}_{-0.26}$, $b = 54.24^{+0.49}_{-0.14}$ and $\log\sigma_{\mathrm{int}} = -1.61^{+0.29}_{-0.17}$. The best-fit correlation and corresponding 3σ intrinsic scattering region are presented in Supplementary Data Fig. 3c. To maintain consistency with the calculation method of the sample, we estimate the rest-frame lag of GRB 230307A between rest-frame energy bands 100–150 and 200–250 keV to be $1.6^{+1.4}_{-1.2}$ ms. Assuming the redshift to be 0.065, the isotropic peak luminosity of GRB 230307A can be calculated as $L_{\gamma,\mathrm{iso}} = 4.64^{+0.09}_{-0.08} \times 10^{51}$ erg s^{-1} based on the spectral fits to GECAM S-II. Then we place both GRB 230307A and GRB 211211A on the $L_{\gamma,\mathrm{iso}} - \tau_{\mathrm{z}}$ diagram. As illustrated in the Supplementary Data Fig. 3c, GRB 230307A and GRB 211211A exhibit significant deviations from the anti-correlation of type II GRBs. Specifically, their perpendicular distances Δ from the anti-correlation are approximately $8.7\sigma_{\mathrm{int}}$ and $7.4\sigma_{\mathrm{int}}$, respectively. Notably, despite being long-duration GRBs, they are intermingled with other type I GRBs. We also note that, if the redshift is 3.87[13], the extremely high peak luminosity of GRB 230307A would make it a significant outlier in the known sample of GRBs.

6 Fit of multi-wavelength flux light curves

On the basis of detailed spectral fitting to the time-resolved spectra of LEIA S-II and GECAM S-II, we calculate the energy flux for each time slice and construct the multi-wavelength flux light curves for six energy bands. These six energy bands, including 0.5–4, 15–30, 30–100, 100–350, 350–700, and 700–2000 keV, are set by referencing the effective energy ranges of the LEIA, GECAM-B, and GECAM-C. It is noteworthy that, during the last four time slices of GECAM S-II, only the high gain data from GECAM-C and GECAM-B exhibit effective spectra that are significantly above the background level, while the low gain data from GECAM-B are already close to the background level and do not provide effective spectral information, which leads to insufficient confidence in determining flux values in the energy bands above 350 keV. Therefore, based on the Bayesian posterior probability distribution generated by *Multinest*[49, 50, 51, 52], we provide the 3σ upper limits of flux for 350–700 and 700–2000 keV in the last four time intervals. The multi-wavelength flux light curves are represented in Fig. 2a. All these flux light curves display multi-segment broken power law features. To fit these features, we introduce multi-segment SBPL functions.

In general, smoothly connected functions in a logarithmic-logarithmic scale can be expressed as

$$F = (F_{\rm l}^{-\omega} + F_{\rm r}^{-\omega})^{-1/\omega}, \tag{11}$$

where $F_{\rm l}$ and $F_{\rm r}$ are the functions located on the left and right sides respectively, and ω describes the smoothness. When both of $F_{\rm l}$ and $F_{\rm r}$ are power law functions, a two-segment SBPL function can be obtained as

$$F_{12} = (F_1^{-\omega_1} + F_2^{-\omega_1})^{-1/\omega_1}, \tag{12}$$

where $F_1 = A(t/t_{\rm b1})^{-\alpha_1}$, $F_2 = A(t/t_{\rm b1})^{-\alpha_2}$. The power law slopes before and after the break time $t_{\rm b1}$ are α_1 and α_2, respectively, and A is the normalization coefficient at $t_{\rm b1}$. In the case where another break occurs after two-segment SBPL function, a three-segment SBPL function can be expressed as

$$F_{123} = (F_{12}^{-\omega_2} + F_3^{-\omega_2})^{-1/\omega_2}, \tag{13}$$

where $F_3 = F_{12}(t_{\rm b2})(t/t_{\rm b2})^{-\alpha_3}$ describes the third power law function. By extension, we can further expand the three-segment SBPL to include a third break and a forth power law function, namely the four-segment SBPL function:

$$F_{1234} = (F_{123}^{-\omega_3} + F_4^{-\omega_3})^{-1/\omega_3}, \tag{14}$$

where $F_4 = F_{123}(t_{\rm b3})(t/t_{\rm b3})^{-\alpha_4}$ describes the forth power law function.

The fitting process is implemented by the Python module *PyMultinest*[51], a Python interface to the widely-used Fortran nested sampling implementation *Multinest*[49, 50, 51, 52]. We employ χ^2 as the statistical metric to evaluate the likelihood. It should be noted that a dip covering 17–20 s exists in the flux light curves of all energy bands of GECAM. Since the dip is an additional component superimposed on the multi-segment broken power law, the time interval containing the dip is omitted from the fitting procedure and will be examined in a separate analysis, which will be discussed in detail elsewhere[69]. For convenience, we refer to the flux light curves of the six energy bands as LF (0.5–4 keV) and GFi (where i ranges from 1 to 5, representing the five energy bands of GECAM from low to high). We note that LF consists of a shallow power law decay followed by a steeper decline, which is significantly different from GFs, where they include an initial power law rise and several gradually steepening power law decay phases. GF1-3 require the four-segment SBPL functions to describe their features, while GF4 and GF5 can be described using three-segment SBPL functions as the late-time features cannot be constrained due to the last four data points being 3σ upper limits. Interestingly, in the GECAM energy bands, the first three power law segments and the corresponding two break times exhibit clear spectral evolution features. From low to high energy, the power law decay index gradually increases. Additionally, there appears to be a tendency for the break times to shift towards earlier intervals. However, we note that the final breaks in GF1-3 appear to be a simultaneous feature. Such an achromatic break is typically attributed to the geometric effects of the emission region.

To test the simultaneity of the final break and determine its break time, we performed a joint fit to GF1-3. The fitting process is described as follows. We simultaneously use three four-segment SBPL functions to fit GF1, GF2, and GF3 independently but allow the three t_{b3} parameters to degenerate into a common parameter. The statistic of the joint fit is the sum of χ^2 values for GF1-3. The fitting process is also implemented by the Python module *PyMultinest*[51]. The best-fitting parameter values and their 1σ uncertainties are presented in Supplementary Data Table 4. Supplementary Data Fig. 4a exhibits the corresponding corner plot of posterior probability distributions of the parameters for the joint fit, where all the parameters are well constrained, and the common parameter t_{b3} is also well constrained to be $84.3^{+1.2}_{-1.2}$ s. It should be noted that for visual clarity, only the posterior probability distributions of the achromatic break time (t_{b3}) and its previous decay indices ($\hat{\alpha}_3$) are displayed. For GF4, if t_{b3} is fixed at 84.3, the flux upper limits of the last four time intervals can constrain the lower limit of $\hat{\alpha}_4$ to be 6.5.

7 Curvature effect

The "curvature effect" refers to the phenomenon of photons arriving progressively later at the observer from higher latitudes with respect to the line of sight[70, 71]. It has been proposed that this effect plays an important role in shaping the decay phase of the light curve after the sudden cessation of the GRB's emitting shell[72, 73]. By assuming a power law spectrum with spectral index $\hat{\beta}$ for the GRB, the most straightforward relation of the curvature effect can be given as $\hat{\alpha} = 2 + \hat{\beta}$[70, 71, 74], where $\hat{\alpha}$ and $\hat{\beta}$ are the temporal decay index and spectral index in the convention $F_\nu \propto t^{-\hat{\alpha}}\nu^{-\hat{\beta}}$, respectively. If the aforementioned assumptions are released, and the intrinsically curved spectral shape and strong spectral evolution are taken into account, the above relationship is no longer applicable[75, 76]. Nevertheless, for a narrow energy band, the intrinsically curved spectrum can be approximated, on average, by a power law, and the time-dependent $\hat{\alpha}$ and $\hat{\beta}$ still approximately follow the relation $\hat{\alpha}(t) = 2 + \hat{\beta}(t)$[76].

The multi-wavelength flux light curves of GRB 230307A exhibit an initial rise and subsequent power law decay phases that gradually become steeper. To test whether the decay phases are dominated by the curvature effect, we compare the time-dependent $\hat{\alpha}(t)$ and $2 + \hat{\beta}(t)$ in each narrow energy band. Here, $\hat{\alpha}(t)$ is obtained by assuming the trigger time T_0 as the time zero point and numerically calculating $-\Delta\log F/\Delta\log t$ of the best-fitting SBPL functions, while $\hat{\beta}(t)$ is the average spectral index $-\Delta\log F_\nu/\Delta\log E$ calculated in the corresponding narrow energy band based on the spectral fitting results for each time slice of LEIA S-II or GECAM S-II. Fig. 2c displays the time-dependent $\hat{\alpha}(t)$ and $2 + \hat{\beta}(t)$ for each energy band. We note that the power law decay indices of the segments between the second and third breaks (see t_{b2} and t_{b3} in Supplementary Data Table 4) of GECAM multi-wavelength flux light curves are consistent with the prediction of the curvature effect, implying that the jet's emitting shell stops shining at t_{b2} ($\sim$ 23 s) and then high-latitude emission dominates the prompt emission. On the contrary, LEIA flux light curve is in a shallower decay

phase, with a decay index much lower than the prediction of the curvature effect. Such a behavior suggests that the soft X-ray emission detected by LEIA is intrinsic to the central engine, not related to the narrow jet but consistent with the emission due to direct dissipation of the magnetar wind.

As stated above, the relation $\hat{\alpha}(t) = 2 + \hat{\beta}(t)$ is only an approximation for an intrinsically curved spectrum. To more precisely test the curvature effect, we also employ another more sophisticated approach. The curvature effect postulates that the spectrum in the comoving frame is the same for different latitudes with respect to the line of sight. In order to maintain consistency with the observed spectral shape, we consider a time-dependent cutoff power law spectrum in the observer frame with the form

$$N(E,t) = A(t)\Big(\frac{E}{100\ \mathrm{keV}}\Big)^{\alpha}\exp\Big[-\frac{(2+\alpha)E}{E_{\mathrm{p}}(t)}\Big], \tag{15}$$

where $\alpha = -(\hat{\beta}+1)$ is the photon spectral index, $E_{\mathrm{p}}(t)$ is time-dependent peak energy, and $A(t)$ is the time-dependent normalization parameter in units of photons $\mathrm{cm}^{-2}\ \mathrm{s}^{-1}\ \mathrm{keV}^{-1}$. The curvature effect predicts $E_{\mathrm{p}}(t) \propto t^{-1}$ and $N(E_{\mathrm{p}}, t) \propto t^{-1}$[70]. Considering the t_0 effect[72, 73], one can get[76]

$$E_{\mathrm{p}}(t) = E_{\mathrm{p,c}}\Big(\frac{t-t_0}{t_{\mathrm{c}}-t_0}\Big)^{-1}, \tag{16}$$

and

$$A(t) = A_{\mathrm{c}}\Big(\frac{t-t_0}{t_{\mathrm{c}}-t_0}\Big)^{\alpha-1}, \tag{17}$$

where t_0 refers to the time zero point of the pulse being considered for the curvature effect, t_{c} is the epoch when the curvature effect begins, while $E_{\mathrm{p,c}}$ and A_{c} denote the peak energy and normalization parameter at t_{c}, respectively. On the basis of Eqs. 15, 16 and 17, the model spectrum predicted by the curvature effect can be determined at any time t once the parameter set $\{\alpha,\ E_{\mathrm{p,c}},\ A_{\mathrm{c}},\ t_0,\ t_{\mathrm{c}}\}$ is given. In order to compare with the observed spectra and test for consistency with the curvature effect, we fix t_{c} at 23 s based on the results of the aforementioned approximation method and vary the other parameters. Then, we fit the model $N(E,\ t,\ \mathcal{P})$, where $\mathcal{P} = \{\alpha,\ E_{\mathrm{p,c}},\ A_{\mathrm{c}},\ t_0\}$, to the GECAM S-II spectra within the time range spanning from t_{b2} to t_{b3}. After achieving a successful fit with statistically acceptable reduced statistic PGSTAT/dof ~ 1, we present the corner plot of the posterior probability distributions of the free parameters and the comparison between the data and the model in Supplementary Data Fig. 4b and 4c, respectively. We note that the best-fitting parameters of α, $E_{\mathrm{p,c}}$ and A_{c} agree with the fitting parameters obtained through empirical model fitting in Supplementary Data Table 2. Additionally, the time zero point is constrained to the trigger time T_0, which is the prerequisite for the aforementioned approximation method. This indicates that the entire prompt emission is the broad pulse under consideration for the curvature effect. According to Ref.[77, 78], such a broad pulse corresponds to the "slow variability" component associated with the central engine activity, while the "fast variability" component with multiple sharp pulses superposed on the slow component can be attributed to the presence of mini-jets resulting from turbulent reconnection within the emission region. We note that despite our efforts to minimize the bin size while performing spectral fitting through data binning, we unavoidably sacrifice temporal information of the "fast variability" component. Nonetheless, it does not affect the overall shape of

the broad pulse. In summary, we successfully conducted a self-consistency check on the existence of curvature effect between approximately 23–84 s in GECAM data using the two methods mentioned above.

8 Fit of spectral energy distribution

The broad-band prompt emission data enables us to examine the spectral energy distribution (SED) from soft X-rays to gamma-rays. We first perform an independent spectral fitting on the LEIA S-III spectra, by employing the PL model with Galactic absorption ($N_{\rm H} = 1.26 \times 10^{21}$ cm^{-2}) and intrinsic absorption ($N_{\rm H} = 3.92 \times 10^{21}$ cm^{-2}), to derive the theoretical spectra in the LEIA band. We then perform independent spectral fitting on the GECAM S-III spectra, utilizing the same seven empirical models applied to the GECAM S-II spectra. The spectral fitting results of the preferred models, along with the corresponding fitting statistics, are presented in Supplementary Data Table 3. The model-predicted SEDs derived from the spectral fittings at different time intervals are displayed in Fig. 2b.

As shown in Fig. 2b, it is evident that during the first eight time intervals, the extrapolations of the LEIA spectra deviate strongly from the GECAM spectra. Similarly, the extrapolations of the GECAM spectra deviate significantly from the LEIA spectra, regardless of whether the GECAM spectra exhibit breaks at low energies. Despite conducting the spectral fittings by treating the intrinsic absorption as a free parameter, we found that the inconsistency between the LEIA and GECAM spectra still exists. Such inconsistency suggests the presence of a distinct radiation process that dominates the LEIA band. As we can see, during the last two time intervals (after 76 s), the LEIA and GECAM spectra can be jointly fitted well using the single CPL model. This suggests that during this time interval, the high-energy emission component gradually diminishes and that the entire spectrum is dominated by the emergent new component LEIA observed throughout.

In order to better justify the superposition between two emission components, we present a possible model for joint SED fitting by superimposing two spectral models that respectively account for the high-energy (jet) and low-energy (magnetar) spectral components. Below we describe these two spectral models:

- High-energy spectral model: Firstly, we notice that, in Fig. 2b, the natural extrapolations of the GECAM spectral models either partially or entirely over-predict the soft X-ray component. This implies that the contribution of the high-energy emission component to the soft X-ray spectrum should not be the simple extrapolation of the GECAM spectral model into the LEIA band, but rather requires a spectral break between 4–15 keV to reduce the contribution from the high-energy spectral component. However, the energy of such a spectral break and the power law index below the break are relatively arbitrary and cannot be constrained well by

data with the absence of the data between 4–15 keV. On the other hand, considering that the prompt emission of GRBs is typically interpreted as synchrotron radiation originating from a group of non-thermal electrons, one can introduce a synchrotron self-absorption frequency break at $\nu_{\rm sa}$, which is physically expected. The power law index below $E_{\rm sa}$ (i.e. $h\nu_{\rm sa}$) is predicted to be $N(E) \propto E$ or $\propto E^{3/2}$, depending on if a 2SBPL or CPL spectrum is observed in the high-energy band for our case[79]. Therefore, we employ two physically-motivated new spectral models to describe the high-energy (jet) spectral component, namely $\rm PL_{sa}$-2SBPL and $\rm PL_{sa}$-CPL, where $\rm PL_{sa}$ represents the new hard power law segment below $E_{\rm sa}$, and the smooth connection between $\rm PL_{sa}$ and 2SBPL (or CPL) occurs at $E_{\rm sa}$ in accordance with Eq. 11.

- Low-energy spectral model: Considering that the low energy component dominates the entire spectrum at late times, we utilize the CPL model fitted to the tenth joint SED as the template, with the cutoff energy fixed at 53 keV (obtained from the tenth SED's CPL model fit, where the magnetar component dominates, see Supplementary Data Fig. 5 and Supplementary Data Table 3), while allowing the index and normalization parameters to vary freely.

During the joint SED fitting, taking into account the calibration uncertainties of less than 10% for both LEIA and GECAM, we therefore introduce two additional free calibration constants with a prior that allows for 10% deviation. The fitting results, along with the corresponding fitting statistics, are presented in Supplementary Data Table 3. The comparisons between the observed and model-predicted count spectra, as well as the residuals (defined as (data − model)/data error) are depicted in Supplementary Data Fig. 5a. The model-predicted SEDs, along with the high- and low-energy spectral components derived from the spectral fittings at different time intervals, are displayed in Supplementary Data Fig. 5b. The results confirm the successful fit of the model and suggest that the time-dependent SEDs can be interpreted as a superposition of two distinct components: the high-energy component, which diminishes quickly both at later times and at lower energies, and the low-energy component, which is observed consistently by LEIA throughout the observation period.

9 Explanation of multi-wavelength flux light curves

We conduct a smoothly broken power law fit to the multi-wavelength flux light curves (Fig. 2a) and explain each of the segments using our schematic model depicted in Supplementary Data Fig. 6. The jet and magnetar-powered emission processes are displayed separately. The emission of the jet-dominated GRB component undergoes a rise followed by a general trend of decline contributed by photons within the jet core of $\theta_{\rm c} = 1/\Gamma$, where Γ is the Lorentz factor. After the emitting shell terminates, the emission is contributed by photons from higher latitude regions with respect to the line of sight of the observer. The best-fit temporal slopes during this phase ($t_{\rm b2}$–$t_{\rm b3}$ in Extended Date Table 4) are consistent with prediction of the high-latitude curvature effect relation $\hat{\alpha}(t) = 2 + \hat{\beta}(t)$

(Fig. 2c). Following the progressive decrease of the high-latitude emission, an achromatic break occurs, signaling the end of the curvature effect. This enables us to estimate the jet opening angle, for the first time, during the prompt emission phase of a GRB. Finally, the light curve drops with an even steeper slope, possibly contributed by some very weak emission from regions beyond the opening angle of the jet that is likely to have no sharp edges. The detailed energy and Lorentz factor profiles beyond the jet edge can be constrained from the data[80]. In contrast, the soft X-ray emission is powered by a presumably more isotropic magnetar wind. The light curve is dominated by the spin-down law with a plateau followed by a shallow decline.

10 Estimation of jet opening angle

The high-latitude emission effect is observed in the multi-wavelength flux light curves of GECAM, starting from the second break ($t_{\rm b2}$) and continuing until the third break ($t_{\rm b3}$), as depicted in Fig. 2 (see also Supplementary Data Table 4). The third break signifies the moment when photons from the outermost layer of the shell, with a radius $R_{\rm GRB}$, reach the observer. The duration of such tail emission can be described by the following relationship[72]:

$$\Delta t_{\rm b} = t_{\rm b3} - t_{\rm b2} = (1+z)\left(\frac{R_{\rm GRB}}{c}\right)\left(\frac{\theta_j^2}{2}\right), \tag{18}$$

where θ_j represents the half opening angle of the jet. By substituting $t_{\rm b3} = 84$ s, $t_{\rm b2} = 23$ s, and assuming a typical radius of $R_{\rm GRB} = 10^{15}$ cm, we can calculate the opening angle of the jet as follows:

$$\theta_{\rm j} = \sqrt{\frac{2c\Delta t_{\rm b}}{(1+z)R_{\rm GRB}}} \approx 3.4^\circ \left(\frac{\Delta t_{\rm b}}{61\ {\rm s}}\right)^{\frac{1}{2}} \left(\frac{R_{\rm GRB}}{10^{15}\ {\rm cm}}\right)^{-\frac{1}{2}}. \tag{19}$$

Such a jet opening angle is generally consistent with that derived from multi-band modeling of the afterglows[81, 82], where the jet break is observed around a few days after the event. However, it is smaller than that reported in another study[83], which used different energy sources for kilonova modeling. The achromatic break observed in our work is due to the edge effect of high-energy photons produced from internal emission, while in the afterglow, it is due to external shocks at a larger radius. However, it is also possible that the jet opening angle measured in the prompt emission could be somewhat different from that measured in the afterglow considering there could be additional angular energy injection at late times from the magnetar wind.

11 Host galaxy

We conducted a search for the host galaxy of GRB 230307A in various public galaxy catalogs and GCN circulars. We estimated the chance coincidence probability P_{cc}[84] for the most promising candidates, which are listed below.

(1) Large Magellanic Cloud (LMC): The LMC is the nearest and brightest galaxy to the Milky Way, located at a distance of 49 kpc. GRB 230307A is 8.15 degrees away from the center of the LMC, corresponding to a physical separation of 7 kpc, and it is situated on the edge of the Magellanic Bridge. The surface density of galaxies, typically used for estimating chance coincidence probabilities, is not applicable to the LMC due to its brightness. Instead, we consider a surface density of galaxies as bright as the LMC, which is $\sigma = 1/41252.96$ deg^{-2}. Using a half-light radius of $r_{50} = 2.2$ deg[85], we estimate the chance coincidence probability P_{cc} to be 0.006. However, its physical properties are not fully consistent with known transients. Firstly, with LMC as the host galaxy, the isotropic energy would be $\sim 9 \times 10^{44}$ erg, much lower than typical GRBs. Secondly, although the isotropic energy is consistent with those of Soft Gamma-ray Repeater (SGR) giant flares, the duration is much longer than the short hard spikes of the giant flares. Furthermore, its large offset with respect to the LMC disk is inconsistent with that of known SGRs. Therefore, we consider this scenario less likely.

(2) Galaxy with a redshift of $z = 3.87$: According to Ref.[13], there is a faint galaxy located 0.2 arcsec away from GRB 230307A with a redshift of $z = 3.87$. We analyzed the JWST/NIRCam images using the official STScI JWST Calibration Pipeline version 1.9.0 and found the galaxy to be fainter than 28.5 mag in the JWST/F070W bands and 27.4 mag in the JWST/F277W band. The estimated P_{cc} using the JWST/F277W magnitude is 0.034, while for the JWST/F070W band, it is estimated to be greater than 0.09. Since the F070W band is closer to the r band, with which the galaxy brightness distribution is produced, the latter estimate is considered more reliable. However, the extremely high energy of the GRB and its inconsistency with known GRB transients in Supplementary Data Fig. 3b and 3c make this scenario less favored.

(3) Galaxy with a redshift of $z = 0.065$[10, 14]: With the half-light radius and r band magnitude from DESI Legacy Survey[86][3], the chance coincidence probability for this galaxy was estimated to be 0.11 (Table 1). Although not statistically significant, the redshift is more consistent with the physical properties of the GRB. Therefore, we consider this galaxy to be the most likely host. The offset of GRB 230307A from the host galaxy is 29.4", which is 36.6 kpc in redshift 0.065. As presented in Supplementary Data Fig. 3d, the offset is consistent with those of type I GRB, and larger than type II GRBs. Specifically, the offset is approximately $1(1.3)\sigma$ away from the type I track and $2.4(8.9)\sigma$ away from the type II track in logrithmic (linear) space.

[3]https://www.legacysurvey.org/dr10/catalogs/

Moreover, we explore other possible host galaxies in DESI Legacy Survey. For objects within 5 arcmin of GRB 230307A, we exclude stars with detected parallax in Gaia, and then calculate the chance coincidence probability with the half-light radius and r band magnitude in the catalog. It turns out that the galaxy with a redshift of $z = 0.065$ has the lowest $P_{\rm cc}$, while others have $P_{\rm cc} > 0.2$ or more.

12 LEIA data compared with other X-ray and optical observations

To investigate whether the LEIA observation is consistent with the early X-ray afterglow, we have incorporated the predicted afterglow model from late-time multi-wavelength observations[81], as illustrated in Fig. 3b. The estimated X-ray flux of the afterglow at approximately a few hundred seconds in the 0.5–4 keV range is $\sim 10^{-11}$ erg cm^{-2} s^{-1}, which is more than two orders of magnitude below the observed flux. We also note that the power law index of the LEIA light curve in the decaying phase is around -2, which differs from the typical value of GRB X-ray afterglows, which is around -1.

GRB 230307A was also simultaneously detected by TESS[87] in the optical during the prompt emission and the following afterglow phases. We calculated the X-ray to optical flux density ratios at the epochs when the burst was jointly detected in these two energy bands, as illustrated in Supplementary Data Fig. 7. The results show that the flux density ratios at the early epochs are significantly higher than those at later times. The abnormally large ratios during the first two epochs imply that there is an additional component contributing to the early X-ray data. Additionally, the TESS observation shows that the optical afterglow rises to its peak at approximately 0.05 days, which is much longer than the 200-second duration of the LEIA data.

Therefore, the soft X-ray component detected by LEIA is inconsistent with the X-ray afterglow of the GRB.

13 Magnetar Model

We fit the LEIA (0.5–4 keV) light curve of GRB 230307A in the prompt emission phase with both the smoothly broken power law model (Eq. 12) and the magnetar dipole radiation model.

Considering a millisecond magnetar with rigid rotation, it loses its rotational energy through

both magnetic dipole radiation and quadrapole radiation[88, 89, 90, 91, 92], with

$$\dot{E} = I\Omega\dot{\Omega} = -\frac{B_{\rm p}^2 R^6 \Omega^4}{6c^3} - \frac{32GI^2\epsilon^2\Omega^6}{5c^5}, \tag{20}$$

where $\dot{E}$ is the total spin-down rate, $\Omega = 2\pi/P$ is the angular frequency and $\dot{\Omega}$ its time derivative, I is the moment of inertia, $B_{\rm p}$ is the dipolar field strength at the magnetic poles on the NS surface, R is the NS radius, and ϵ is the ellipticity of the NS. In the following calculations, we use the values $I = 3.33 \times 10^{45}$ g cm^{-2}, $R = 1.2 \times 10^6$ cm, based on the Equation of State GM1 as discussed in Ref.[93], and $\epsilon = 10^{-4}$. The electromagnetic emission is determined by the dipole spin-down luminosity $L_{\rm sd}$, i.e.,

$$L_{\rm X}(t) = \eta L_{\rm sd} = \frac{\eta B_{\rm p}^2 R^6 \Omega^4(t)}{6c^3}, \tag{21}$$

where η is the efficiency of converting the dipole spin-down luminosity to the X-ray luminosity. The X-ray luminosity is derived from the observation assuming isotropic emission. In a more realistic situation, the X-ray emission may not be isotropic, and a factor $f_{\rm b}$ (assumed to be 0.1) is introduced to account for this correction between the isotropic and true luminosity $L_{\rm X}$, i.e.,

$$L_{\rm iso}(t) = \frac{L_{\rm X}(t)}{f_{\rm b}} = \frac{\eta}{f_{\rm b}} \frac{B_{\rm p}^2 R^6 \Omega^4(t)}{6c^3}. \tag{22}$$

We model the unabsorbed luminosity light curves based on Eqs. 20 and 22. The fitting process is performed by using the Markov Chain Monte Carlo code emcee[64]. We employ χ^2 as the statistical metric to evaluate the likelihood. The prior bounds for the free parameters ($\log(B_{\rm p}/{\rm G})$, P_0/ms, $\log \eta$) are set to be $(15, 17)$, $(1, 5)$, and $(-3, -2)$, respectively. The first data point is excluded, as the soft X-ray data possibly suffer the contamination from the jet emission at the very early time. The fitting results are shown in Fig. 3a and Supplementary Data Fig. 8. After subtracting the contribution of the jet component based on the SED modeling represented in Supplementary Data Fig. 5b, we calculate the flux of the magnetar component in 0.5–4 keV and fit the light curve using both the smoothly broken power law model and the magnetar dipole radiation model, as shown in Supplementary Data Fig. 5c. The best-fit slopes and magnetar parameters are consistent with those derived in Fig. 3a within 1σ uncertainties.

Fast X-ray transients with light curves characteristic of spin-down magnetars have been identified previously in the afterglow of some short GRBs, as well as in a few events without associated GRB such as CDF-S XT2, which are believed to originate from compact star mergers. We compare the X-ray luminosity light curve of GRB 230307A with internal plateaus in the X-ray afterglows of short GRBs with redshifts and in CDF-S XT2. The extended emissions and the afterglow data are retrieved from the BAT/XRT lightcurve repository[94, 95, 96] and corrected to the 0.5–4 keV band assuming an absorbed power law spectrum. Such a correction is also made for the luminosity of CDF-S XT2, using power law indices $\Gamma_1 = 1.45$ and $\Gamma_2 = 2.67$ before and after the break time at 2.3 ks[97].

When a magnetar is formed through the merger of binary neutron stars, it is anticipated that there is additional injection of energy into the ejecta that powers the optical/near-IR kilonova. Early modeling assumed an isotropic magnetic wind and a relatively high spin-down injection efficiency, which predicts a kilonova luminosity that is boosted by a factor of ten or even more[98]. Recent more detailed modeling suggests that for a Poynting flux dominated wind, the heating efficiency after shock crossing is very low[99]. Furthermore, relativistic magnetohydrodynamic simulations suggest that the magnetar wind is anisotropic due to collimation towards the jet direction. The energy injection into the kilonova ejecta is reduced by a factor of up to 10[100].For this burst, the observed kilonova could be consistent with the central engine of a magnetar if the magnetar wind is Poynting flux dominated without significant dissipation. It is worth noticing that the spectral features predicted from the kilonova model is missing from the late-time data (Supplementary Data Fig. 4 of Ref.[101]). This suggests an additional heating source other than radioactive heating, which is consistent with energy injection from a long-lived magnetar.

Although various theories of engine injection have been proposed for both long and short GRBs, such as fallback accretion and refreshed shocks, we have found that these models are not fully consistent with the light curve observed by LEIA. In the case of the BH engine, fallback accretion involves a long-lived disk that would eventually evaporate quickly, causing the jet engine to cease abruptly[102]. This model predicts long-lasting activities with luminosity decaying as $\sim t^{-40/9}$, which is much steeper than the observed slope from the LEIA data[103]. For the refreshed shocks scenario, the decay slope would be flatter, similar to that observed in GRB afterglows (around $t^{-1.5}$). This model has been ruled out due to the inconsistency of the LEIA data with the X-ray afterglow.

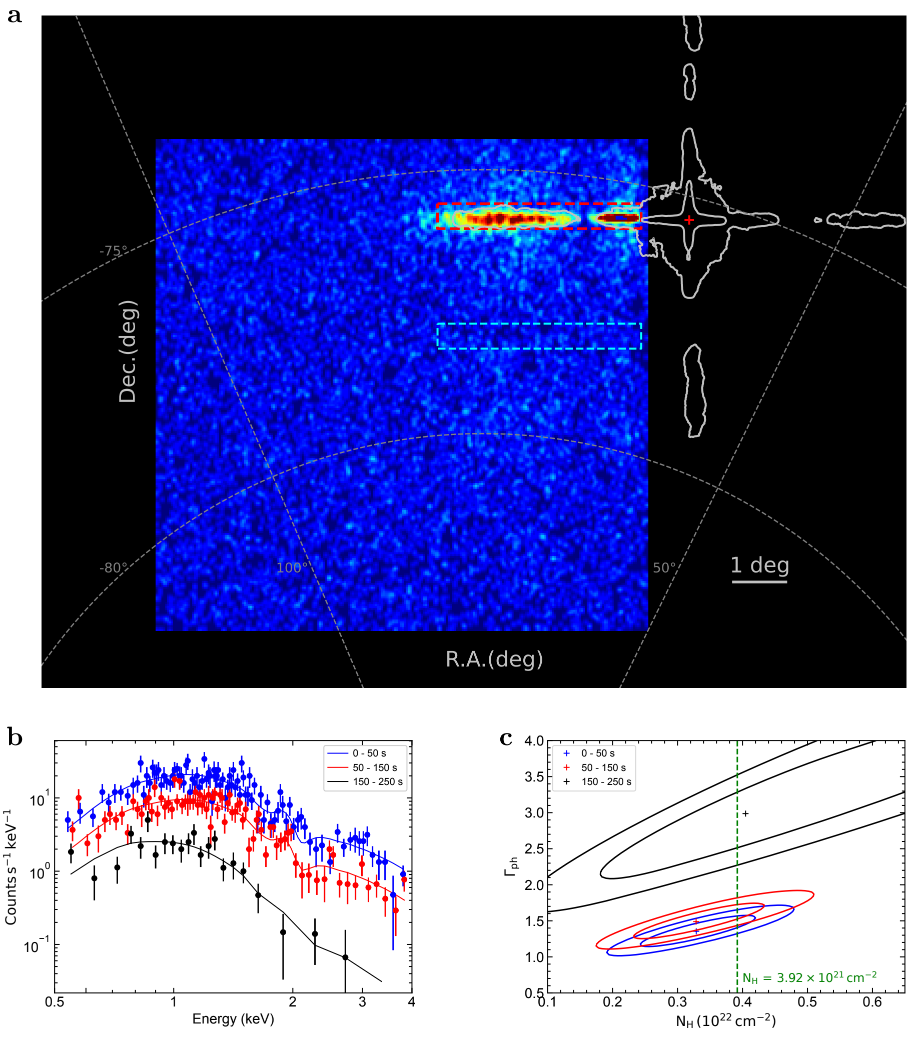

Supplementary Data Fig. 1: **LEIA image and spectra.** **a**, LEIA image. The blue square region represents the quarter of LEIA FoV adjacent to the source. The source with its XRT position denoted by the red cross is located outside of the nominal FoV of LEIA. Therefore only the cruciform arm structure of the point spread function (PSF) was observed, as shown by the brightening regions in the upper right corner. The white solid lines around the source position shows the full PSF profile when the source is detected at this position (the data for generating the PSF profile is taken from on-ground calibration measurements). The regions encircled by red and cyan dash-dotted lines are utilized in the extraction of the source and background photons, respectively. **b**, LEIA observed and model-predicted spectra in three time segments (LEIA S-I). **c**, Best-fit values of photon index $\Gamma_{\rm ph}$ and hydrogen column density $N_{\rm H}$ and 1σ, 2σ confidence contours.

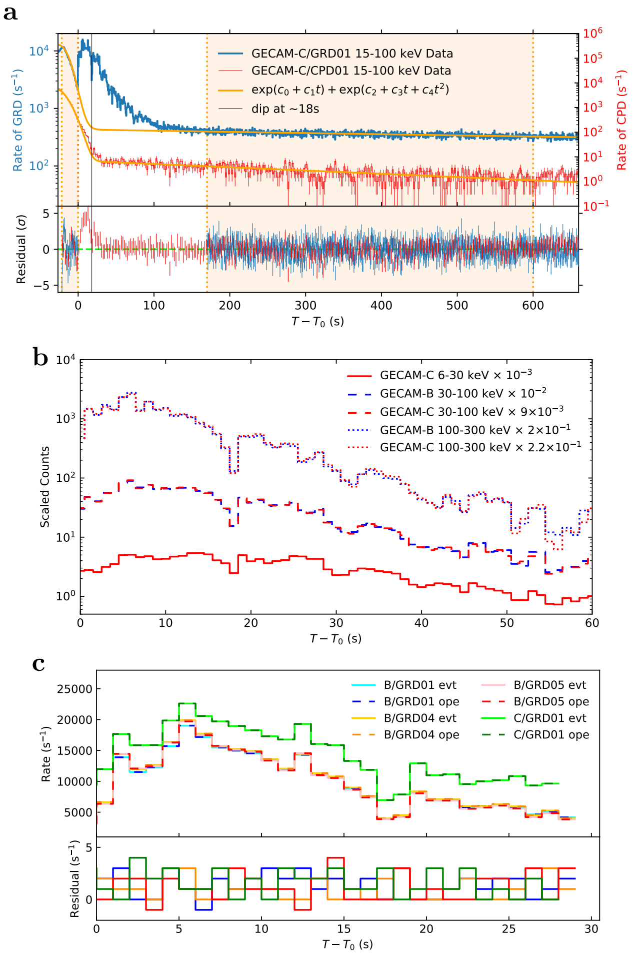

Supplementary Data Fig. 2: **GECAM data reduction.** **a**, The raw light curve of GECAM-C/GRD01 (blue solid line), GECAM-C/CPD01 (red solid line) and the background estimation (orange solid line). The light curve data points within the orange dashed line are used to fit the background model, which is from $T_0 - 21.6$ s to $T_0 - 0.1$ s and $T_0 + 170$ s to $T_0 + 600$ s, then the background estimation can be obtained by interpolating the fitted model over the burst time interval. The fit starting from $T_0 - 21.6$ s is due to data saturation before $T_0 - 21.6$ s caused by the particles in orbit. **b**, Comparison of the net light curves of GECAM-B and GECAM-C. The consistency between these two instruments validates the background estimation of GECAM-C. **c**, Comparison of recorded event rate (evt data, solid line) and processed event rate (ope engineering data, dashed line) of GECAM-B and GECAM-C for GRB 230307A. It is shown that the difference between these two event rates is negligible, demonstrating that both GECAM-B and GECAM-C have no data saturation for GRB 230307A.

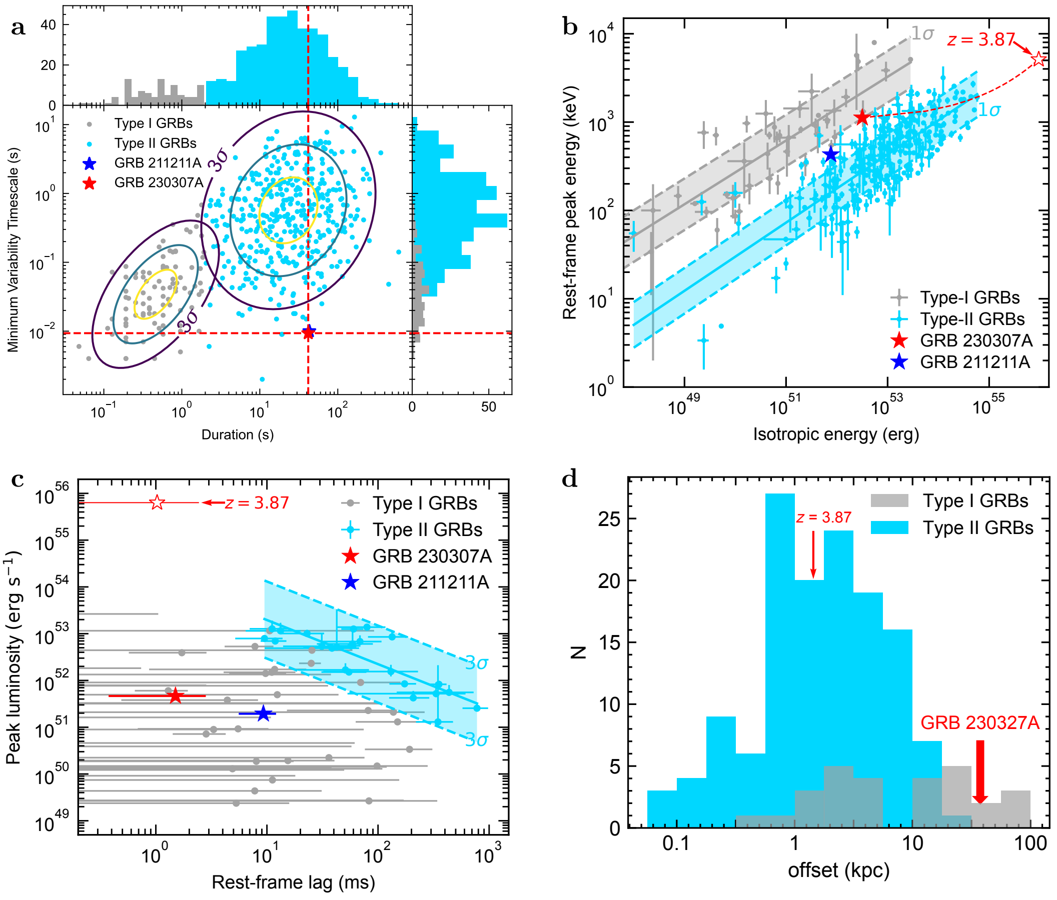

Supplementary Data Fig. 3: **Position of GRB 230307A in various GRB classification schemes.** **a**, The minimum variability timescale versus duration diagram. **b**, The rest-frame peak energy versus isotropic energy correlation diagram. The best-fit correlations and 1σ regions of intrinsic scatter are presented by solid lines and shaded areas, respectively. **c**, The peak luminosity versus rest-frame lag diagram. The best-fit correlation and 3σ region of intrinsic scatter for type II GRBs are presented by cyan line and shaded area, respectively. In **a**–**c**, type I and type II GRBs are represented by grey and cyan solid circles, respectively. GRB 230307A ($z = 0.065$) and GRB 211211A are highlighted by red and blue filled stars, respectively. GRB 230307A ($z = 3.87$) is marked by a red unfilled star. **d**, The distribution of the physical offset of type I and type II GRBs from the center of the host galaxy[104]. GRB 230307A is overplotted in red, showing its consistency with type I GRBs. In all the figures, the cyan and grey colors represent type II and type I GRBs, respectively, with the assumption that all the bursts under each color belong to their physical categories, namely, with massive star core collapse and compact star merger origins, respectively. All error bars on data points represent their 1σ confidence level.

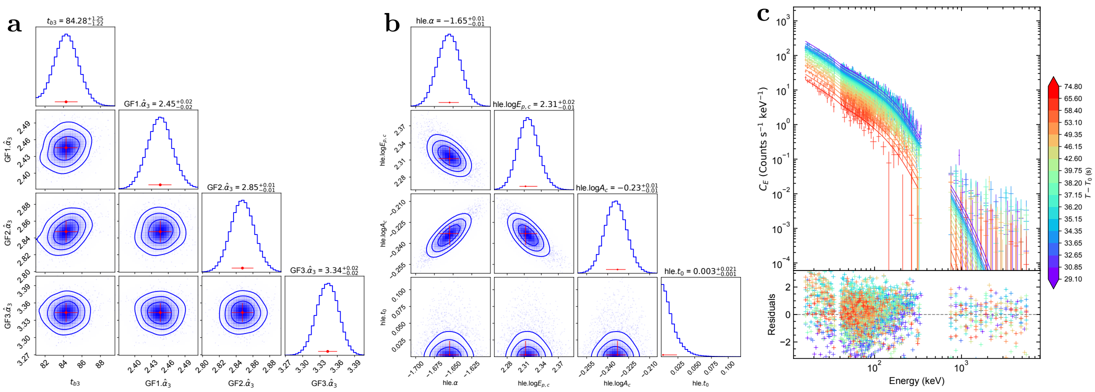

Supplementary Data Fig. 4: **The SBPL model and curvature effect model fits to observed GECAM data. a**, The corner plot of the posterior probability distributions of the parameters for the global SBPL fit to GECAM multi-wavelength (including 15–30, 30–100 and 100–350 keV, denoted by GF1, GF2 and GF3, respectively) flux light curves. It should be noted that for visual clarity, only the achromatic break time ($t_{\rm b3}$) and its previous decay indices ($\hat{\alpha}_3$) are displayed. The red error bars represent 1σ uncertainties. **b**, The corner plot of the posterior probability distributions of the parameters of curvature effect model (denoted by "hle") for the fit to the GECAM S-II spectra. The red error bars represent 1σ uncertainties. **c**, The comparison between observed (points) and model-predicted (lines) count spectra as well as the residuals (pluses) based on the fit of curvature effect model to the GECAM S-II spectra.

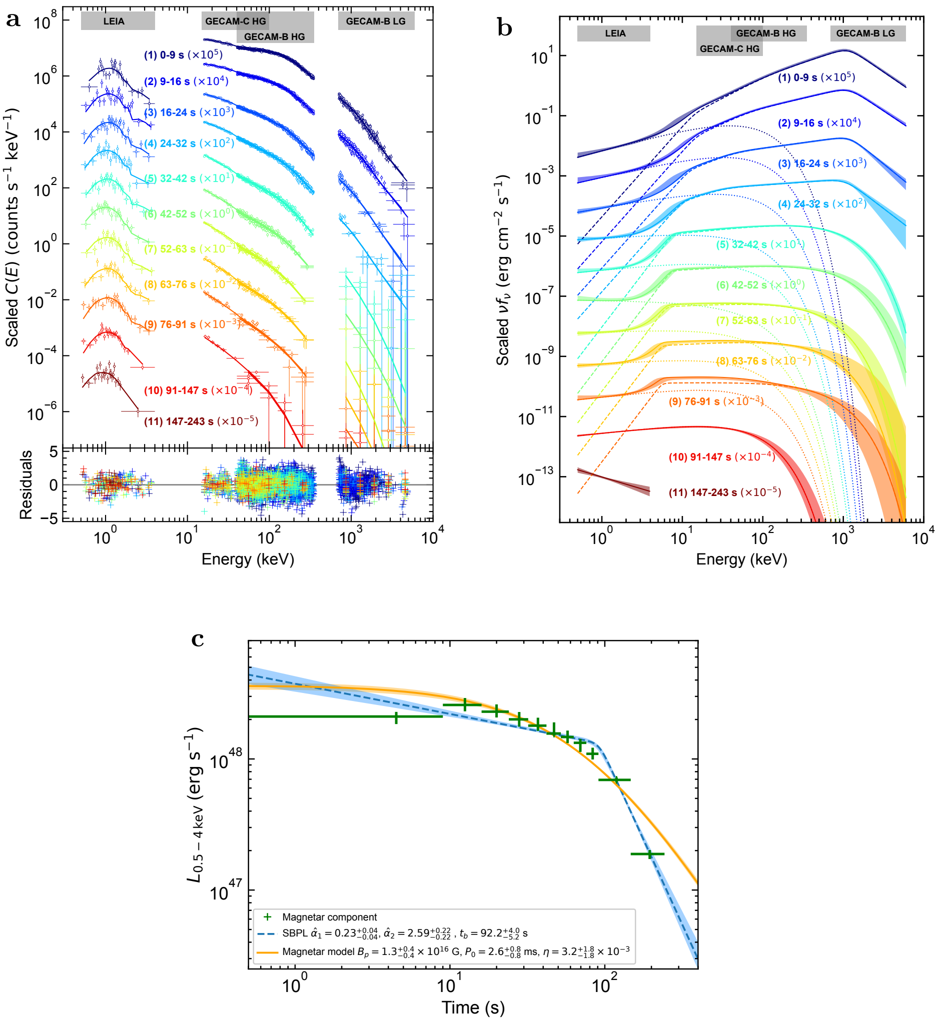

Supplementary Data Fig. 5: **Fit of spectral energy distribution.** **a**, The comparison between observed (points) and model-predicted (solid lines) count spectra as well as the residuals (pluses) based on the joint spectral fittings of LEIA S-III and GECAM S-III. **b**, The model-predicted SEDs derived from the joint spectral fittings of LEIA S-III and GECAM S-III. The high-energy (jet) and low-energy (magnetar) spectral components are represented with the dashed and dotted lines, respectively. The superimposed models are shown with the solid lines. **c**, The X-ray light curve of the magnetar component after the subtraction of the prompt emission. The green data are calculated from the low-energy CPL modelled magnetar-only component. The yellow and blue dashed lines represent the magnetar dipole radiation model and smoothly broken power law model fitted to those calculated magnetar-only data. All error bars and shaded areas represent 1σ uncertainties.

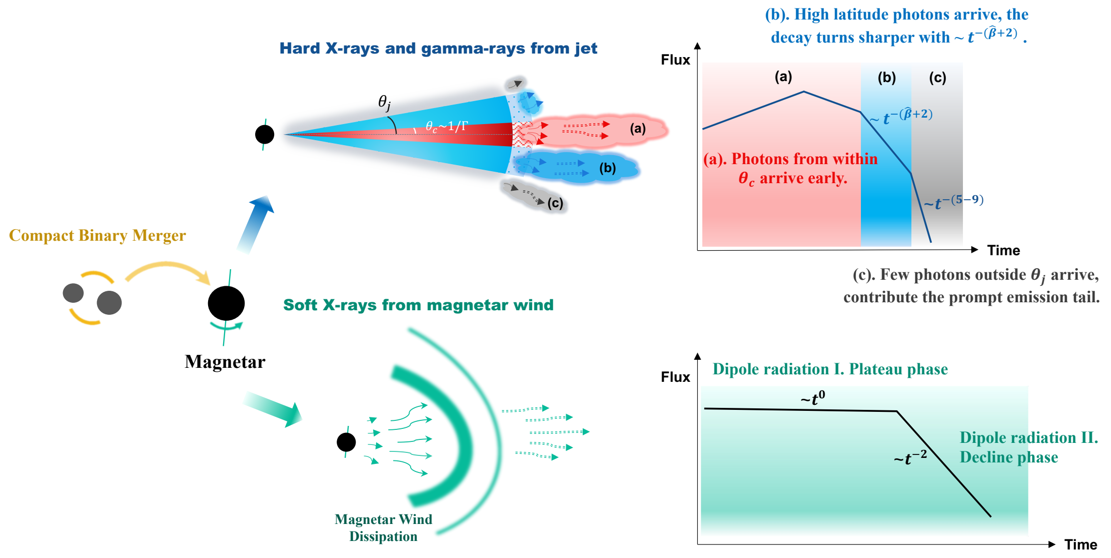

Supplementary Data Fig. 6: **Schematic diagram of the two-component prompt emission of GRB 230307A.** GRB 230307A is suggested to be powered by a millisecond magnetar resulting from a compact binary star merger. In such a scenario, the hard X-rays and gamma-ray are powered by a narrow jet. The early bright emission of the light curve is mainly contributed by photons from the jet within a cone of $\theta_c \sim 1/\Gamma$ at the emission radius $R_{\rm GRB}$ (Phase a). When the jet ceases, photons from higher-latitude parts arrive at later times, resulting in a decay in the light curve with a slope consistent with the curvature effect prediction (Phase b). After an achromatic break signaling the end of the curvature effect, the light curve decays with an even steeper slope. This enables us to estimate the jet opening angle, for the first time, during the prompt emission phase of a GRB. The tail of the prompt emission is possibly contributed by some weak emission from regions beyond the jet opening angle (Phase c). In contrast, the soft X-ray emission is powered by a presumably more isotropic magnetar wind. The light curve is consistent with prediction of the spin-down law with a plateau followed by a shallow decline. All the light curves are displayed in logarithmic coordinate space.

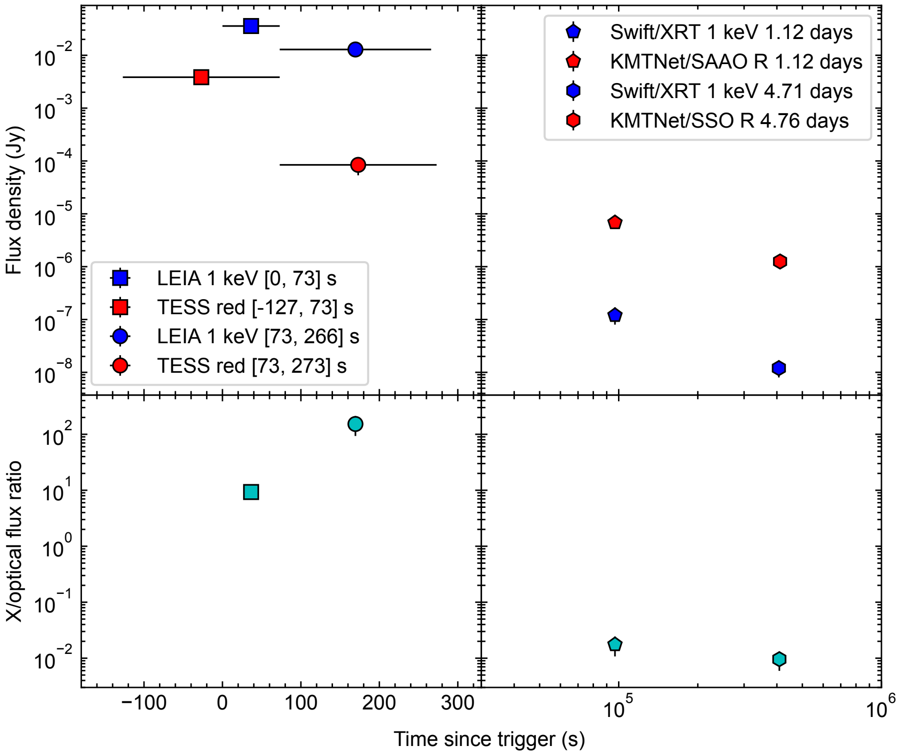

Supplementary Data Fig. 7: **The flux density ratios between X-ray and optical bands for GRB 230307A**. GRB 230307A was simultaneously detected in the X-ray (blue markers) and optical (red markers) bands during the prompt emission (LEIA and TESS[87]) and the following afterglow phases (e.g., Swift/XRT and KMTNet[81]). The flux density ratios during the early prompt emission phase are more than three orders of magnitude higher than those during later times. The abnormally large ratios during the first two epochs imply that there is an additional component contributing to the early X-ray data. This provides strong evidence that the soft X-ray observations from LEIA do not originate from the afterglow emission.

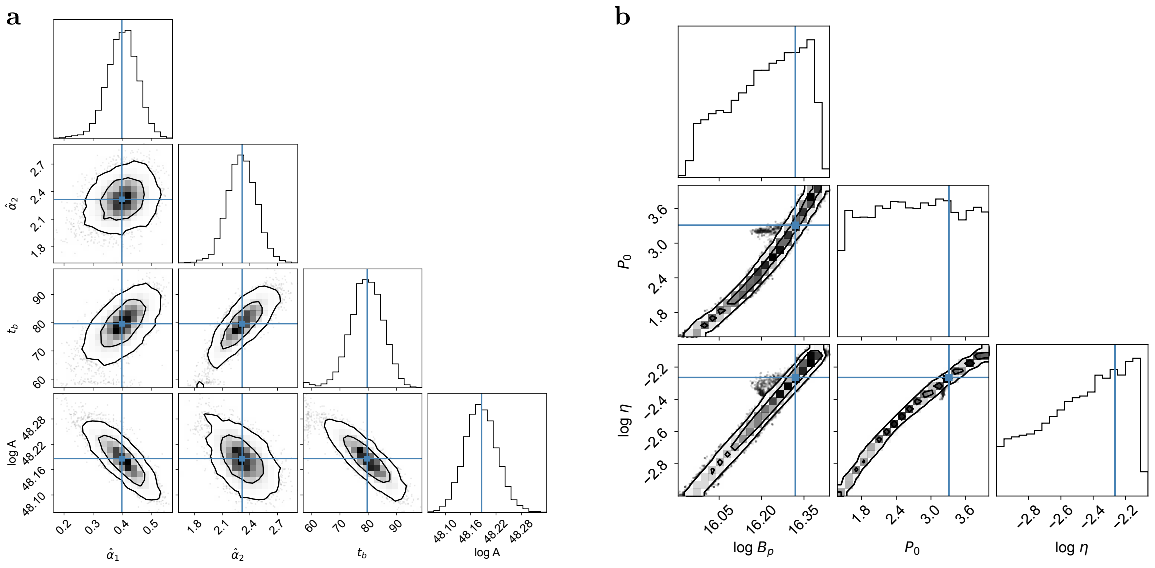

Supplementary Data Fig. 8: **Corner plots of the posterior probability distributions of the parameters for the fit of the X-ray light curve (0.5–4 keV).** **a**, Fit with the smoothly broken power law model. **b**, Fit with the magnetar dipole radiation model.

Supplementary Data Table 1: Spectral fitting results and corresponding fitting statistics for LEIA S-II spectra. All errors represent the 1σ uncertainties.

t_1 (s)	t_2 (s)	$\Gamma_{\rm ph}$	Normalization (photons cm^{-2} s^{-1} keV^{-1})	Unabsorbed flux (erg cm^{-2} s^{-1})	CSTAT/dof
0	6	$0.99^{+0.36}_{-0.35}$	$29.65^{+5.01}_{-4.54}$	$1.68^{+0.33}_{-0.27} \times 10^{-7}$	7.64/6
6	9	$1.15^{+0.36}_{-0.35}$	$70.88^{+13.87}_{-12.07}$	$3.60^{+0.55}_{-0.46} \times 10^{-7}$	15.87/7
9	13	$1.43^{+0.29}_{-0.28}$	$66.73^{+9.08}_{-8.29}$	$2.87^{+0.38}_{-0.33} \times 10^{-7}$	9.67/9
13	17	$1.03^{+0.24}_{-0.24}$	$52.53^{+7.45}_{-6.91}$	$2.89^{+0.38}_{-0.34} \times 10^{-7}$	3.11/9
17	21	$0.96^{+0.30}_{-0.29}$	$44.93^{+8.22}_{-7.29}$	$2.58^{+0.39}_{-0.35} \times 10^{-7}$	9.95/7
21	25	$1.38^{+0.33}_{-0.33}$	$55.87^{+8.51}_{-7.79}$	$2.43^{+0.37}_{-0.31} \times 10^{-7}$	10.01/7
25	29	$1.80^{+0.33}_{-0.32}$	$58.68^{+8.06}_{-7.40}$	$2.11^{+0.28}_{-0.24} \times 10^{-7}$	7.77/6
29	34	$1.59^{+0.30}_{-0.29}$	$50.42^{+6.52}_{-6.02}$	$2.00^{+0.27}_{-0.23} \times 10^{-7}$	6.36/8
34	39	$1.30^{+0.27}_{-0.26}$	$48.26^{+6.84}_{-6.24}$	$2.23^{+0.28}_{-0.25} \times 10^{-7}$	1.75/9
39	45	$1.54^{+0.34}_{-0.32}$	$40.59^{+5.57}_{-5.11}$	$1.64^{+0.24}_{-0.21} \times 10^{-7}$	6.32/8
45	50	$2.10^{+0.41}_{-0.39}$	$52.03^{+7.14}_{-6.68}$	$1.68^{+0.23}_{-0.20} \times 10^{-7}$	7.58/7
50	55	$1.30^{+0.26}_{-0.26}$	$47.19^{+6.43}_{-6.31}$	$2.20^{+0.28}_{-0.25} \times 10^{-7}$	18.18/8
55	61	$1.76^{+0.39}_{-0.38}$	$41.07^{+6.38}_{-5.77}$	$1.50^{+0.22}_{-0.18} \times 10^{-7}$	2.88/6
61	68	$1.39^{+0.28}_{-0.27}$	$35.74^{+4.97}_{-4.57}$	$1.57^{+0.20}_{-0.18} \times 10^{-7}$	9.96/9
68	76	$1.23^{+0.46}_{-0.43}$	$25.73^{+5.33}_{-4.73}$	$1.24^{+0.23}_{-0.19} \times 10^{-7}$	12.58/7
76	84	$1.30^{+0.31}_{-0.30}$	$25.48^{+3.78}_{-3.49}$	$1.18^{+0.18}_{-0.15} \times 10^{-7}$	5.08/7
84	92	$1.49^{+0.31}_{-0.31}$	$29.16^{+4.00}_{-3.67}$	$1.21^{+0.18}_{-0.15} \times 10^{-7}$	4.26/7
92	103	$1.40^{+0.34}_{-0.32}$	$19.38^{+2.71}_{-2.48}$	$8.47^{+1.40}_{-1.17} \times 10^{-8}$	3.55/7
103	116	$2.02^{+0.38}_{-0.36}$	$18.84^{+2.54}_{-2.36}$	$6.23^{+0.88}_{-0.77} \times 10^{-8}$	3.84/7
116	131	$2.14^{+0.35}_{-0.33}$	$16.48^{+2.21}_{-2.06}$	$5.25^{+0.65}_{-0.60} \times 10^{-8}$	5.31/7
131	153	$2.10^{+0.43}_{-0.41}$	$13.27^{+1.77}_{-1.63}$	$4.28^{+0.58}_{-0.48} \times 10^{-8}$	1.95/7
153	187	$2.41^{+0.39}_{-0.38}$	$7.96^{+0.95}_{-0.89}$	$2.37^{+0.29}_{-0.26} \times 10^{-8}$	7.90/8
187	266	$3.16^{+0.37}_{-0.36}$	$4.33^{+0.47}_{-0.45}$	$1.22^{+0.14}_{-0.13} \times 10^{-8}$	7.04/10

Supplementary Data Table 2: **Spectral fitting results and corresponding fitting statistics for GECAM S-I and S-II spectra.** All errors represent the 1σ uncertainties.

t_1 (s)	t_2 (s)	Best model	α_1	$\alpha_2(\alpha)$	β	$\log E_b$ (keV)	$\log E_p(\log E_c)$ (keV)	$\log A$ (photons/cm^2/s/keV)	PGSTAT/dof
**GECAM S-I:**									
1.6	43.2	2SBPL	$-0.92^{+0.05}_{-0.03}$	$-1.274^{+0.005}_{-0.008}$	$-3.85^{+0.03}_{-0.09}$	$1.39^{+0.05}_{-0.03}$	$3.022^{+0.007}_{-0.004}$	$1.83^{+0.04}_{-0.06}$	2209/960
**GECAM S-II:**									
0.0	0.7	CPL	–	$-0.96^{+0.04}_{-0.05}$	–	–	$2.16^{+0.02}_{-0.01}$	$-0.05^{+0.03}_{-0.03}$	854/963
0.7	1.0	CPL	–	$-0.57^{+0.03}_{-0.03}$	–	–	$2.76^{+0.02}_{-0.02}$	$0.02^{+0.01}_{-0.01}$	754/963
1.0	1.4	CPL	–	$-0.66^{+0.03}_{-0.03}$	–	–	$2.72^{+0.02}_{-0.02}$	$0.01^{+0.01}_{-0.01}$	808/963
1.4	1.6	CPL	–	$-0.48^{+0.02}_{-0.03}$	–	–	$3.01^{+0.02}_{-0.01}$	$0.12^{+0.01}_{-0.01}$	886/963
1.6	1.8	SBPL	–	$-0.66^{+0.01}_{-0.03}$	$-3.82^{+0.14}_{-0.33}$	–	$3.01^{+0.02}_{-0.01}$	$1.35^{+0.07}_{-0.03}$	835/962
1.8	2.0	CPL	–	$-0.25^{+0.03}_{-0.03}$	–	–	$3.10^{+0.01}_{-0.01}$	$0.12^{+0.01}_{-0.01}$	954/963
2.0	2.3	SBPL	–	$-0.64^{+0.02}_{-0.02}$	$-4.07^{+0.20}_{-0.33}$	–	$2.86^{+0.02}_{-0.01}$	$1.28^{+0.04}_{-0.04}$	881/962
2.3	2.7	SBPL	–	$-0.69^{+0.01}_{-0.02}$	$-4.22^{+0.31}_{-0.25}$	–	$2.83^{+0.02}_{-0.02}$	$1.33^{+0.05}_{-0.02}$	935/962
2.7	3.2	CPL	–	$-0.73^{+0.02}_{-0.03}$	–	–	$2.82^{+0.02}_{-0.02}$	$-0.04^{+0.01}_{-0.01}$	929/963
3.2	3.4	CPL	–	$-0.42^{+0.02}_{-0.02}$	–	–	$3.08^{+0.01}_{-0.01}$	$0.304^{+0.005}_{-0.006}$	905/963
3.4	3.6	CPL	–	$-0.43^{+0.03}_{-0.02}$	–	–	$3.09^{+0.01}_{-0.02}$	$0.17^{+0.01}_{-0.01}$	955/963
3.6	4.0	CPL	–	$-0.58^{+0.03}_{-0.02}$	–	–	$2.82^{+0.01}_{-0.02}$	$0.06^{+0.01}_{-0.01}$	817/963
4.0	4.3	SBPL	–	$-0.81^{+0.02}_{-0.02}$	$-3.36^{+0.18}_{-0.23}$	–	$2.93^{+0.02}_{-0.01}$	$1.54^{+0.04}_{-0.04}$	929/962
4.3	4.7	CPL	–	$-0.72^{+0.02}_{-0.02}$	–	–	$2.88^{+0.02}_{-0.02}$	$0.04^{+0.01}_{-0.01}$	873/963
4.7	4.9	CPL	–	$-0.54^{+0.02}_{-0.03}$	–	–	$2.98^{+0.02}_{-0.01}$	$0.22^{+0.01}_{-0.01}$	896/963
4.9	5.0	CPL	–	$-0.64^{+0.03}_{-0.04}$	–	–	$2.84^{+0.02}_{-0.02}$	$0.30^{+0.01}_{-0.01}$	773/963
5.0	5.2	CPL	–	$-0.72^{+0.02}_{-0.02}$	–	–	$3.00^{+0.02}_{-0.01}$	$0.30^{+0.01}_{-0.01}$	896/963
5.2	5.4	CPL	–	$-0.65^{+0.02}_{-0.02}$	–	–	$3.01^{+0.01}_{-0.02}$	$0.32^{+0.01}_{-0.01}$	884/963
5.4	5.7	CPL	–	$-0.74^{+0.02}_{-0.02}$	–	–	$2.97^{+0.01}_{-0.02}$	$0.20^{+0.01}_{-0.01}$	869/963
5.7	5.8	CPL	–	$-0.62^{+0.03}_{-0.03}$	–	–	$3.05^{+0.02}_{-0.02}$	$0.25^{+0.01}_{-0.01}$	821/963
5.8	5.9	CPL	–	$-0.52^{+0.03}_{-0.03}$	–	–	$3.13^{+0.02}_{-0.02}$	$0.27^{+0.01}_{-0.01}$	866/963
5.9	6.0	CPL	–	$-0.50^{+0.03}_{-0.02}$	–	–	$3.10^{+0.02}_{-0.02}$	$0.33^{+0.01}_{-0.01}$	956/963
6.0	6.2	CPL	–	$-0.57^{+0.02}_{-0.02}$	–	–	$3.01^{+0.01}_{-0.01}$	$0.36^{+0.01}_{-0.01}$	859/963
6.2	6.3	CPL	–	$-0.56^{+0.03}_{-0.02}$	–	–	$3.09^{+0.02}_{-0.02}$	$0.42^{+0.01}_{-0.01}$	965/963
6.3	6.4	CPL	–	$-0.55^{+0.03}_{-0.03}$	–	–	$3.06^{+0.02}_{-0.02}$	$0.36^{+0.01}_{-0.01}$	916/963
6.4	6.6	CPL	–	$-0.62^{+0.02}_{-0.02}$	–	–	$3.11^{+0.01}_{-0.01}$	$0.293^{+0.005}_{-0.006}$	994/963
6.6	6.7	CPL	–	$-0.53^{+0.03}_{-0.03}$	–	–	$3.12^{+0.02}_{-0.02}$	$0.36^{+0.01}_{-0.01}$	886/963
6.7	6.9	CPL	–	$-0.59^{+0.02}_{-0.02}$	–	–	$3.01^{+0.01}_{-0.01}$	$0.35^{+0.01}_{-0.01}$	925/963
6.9	7.2	CPL	–	$-0.81^{+0.02}_{-0.02}$	–	–	$2.89^{+0.02}_{-0.02}$	$0.09^{+0.01}_{-0.01}$	812/963
7.2	7.4	CPL	–	$-0.75^{+0.03}_{-0.02}$	–	–	$2.91^{+0.02}_{-0.02}$	$0.20^{+0.01}_{-0.01}$	808/963
7.4	7.7	CBAND	$-0.68^{+0.05}_{-0.07}$	$-1.27^{+0.13}_{-0.17}$	–	$2.29^{+0.12}_{-0.13}$	$2.82^{+0.04}_{-0.03}$	$1.61^{+0.10}_{-0.08}$	880/961
7.7	7.9	CSBPL	$-0.43^{+0.67}_{-0.14}$	$-1.00^{+0.05}_{-0.05}$	–	$1.52^{+0.11}_{-0.16}$	$2.89^{+0.03}_{-0.02}$	$1.35^{+0.18}_{-0.86}$	863/961
7.9	8.1	CPL	–	$-0.77^{+0.02}_{-0.02}$	–	–	$3.00^{+0.02}_{-0.02}$	$0.24^{+0.01}_{-0.01}$	882/963
8.1	8.3	CPL	–	$-0.73^{+0.02}_{-0.02}$	–	–	$3.07^{+0.02}_{-0.02}$	$0.27^{+0.01}_{-0.01}$	920/963
8.3	8.4	CPL	–	$-0.75^{+0.03}_{-0.03}$	–	–	$3.07^{+0.02}_{-0.03}$	$0.25^{+0.01}_{-0.01}$	870/963
8.4	8.6	CPL	–	$-0.70^{+0.02}_{-0.02}$	–	–	$3.15^{+0.02}_{-0.01}$	$0.26^{+0.01}_{-0.01}$	976/963
8.6	8.9	CPL	–	$-0.74^{+0.02}_{-0.02}$	–	–	$2.96^{+0.02}_{-0.02}$	$0.19^{+0.01}_{-0.01}$	923/963
8.9	9.1	CPL	–	$-0.77^{+0.02}_{-0.03}$	–	–	$2.95^{+0.02}_{-0.02}$	$0.13^{+0.01}_{-0.01}$	754/963
9.1	9.3	CSBPL	$-0.39^{+0.13}_{-0.26}$	$-1.00^{+0.04}_{-0.11}$	–	$1.54^{+0.31}_{-0.06}$	$2.92^{+0.03}_{-0.04}$	$1.18^{+0.35}_{-0.19}$	782/961
9.3	9.5	CPL	–	$-0.75^{+0.03}_{-0.02}$	–	–	$2.88^{+0.02}_{-0.02}$	$0.25^{+0.01}_{-0.01}$	870/963
9.5	9.7	CPL	–	$-0.87^{+0.02}_{-0.02}$	–	–	$3.04^{+0.02}_{-0.02}$	$0.14^{+0.01}_{-0.01}$	933/963
9.7	10.0	CPL	–	$-0.87^{+0.02}_{-0.02}$	–	–	$3.10^{+0.02}_{-0.02}$	$0.04^{+0.01}_{-0.01}$	863/963
10.0	10.3	2SBPL	$-0.61^{+0.02}_{-0.07}$	$-1.26^{+0.05}_{-0.06}$	$-4.12^{+0.47}_{-0.26}$	$2.03^{+0.08}_{-0.05}$	$3.14^{+0.03}_{-0.04}$	$1.45^{+0.12}_{-0.04}$	973/960
10.3	10.5	CPL	–	$-0.76^{+0.03}_{-0.02}$	–	–	$2.95^{+0.02}_{-0.03}$	$0.15^{+0.01}_{-0.01}$	893/963
10.5	10.7	CSBPL	$-0.14^{+0.52}_{-0.08}$	$-0.96^{+0.06}_{-0.01}$	–	$1.57^{+0.03}_{-0.12}$	$3.00^{+0.01}_{-0.04}$	$0.85^{+0.11}_{-0.70}$	880/961
10.7	10.9	CPL	–	$-0.85^{+0.03}_{-0.02}$	–	–	$2.93^{+0.03}_{-0.02}$	$0.12^{+0.01}_{-0.01}$	796/963
10.9	11.2	CSBPL	$-0.80^{+0.06}_{-0.05}$	$-1.39^{+0.13}_{-0.09}$	–	$2.08^{+0.07}_{-0.13}$	$2.85^{+0.03}_{-0.04}$	$1.73^{+0.08}_{-0.10}$	885/961
11.2	11.4	CBAND	$-0.36^{+0.07}_{-0.17}$	$-1.11^{+0.04}_{-0.08}$	–	$1.99^{+0.13}_{-0.04}$	$2.96^{+0.03}_{-0.03}$	$1.25^{+0.22}_{-0.09}$	849/961
11.4	11.7	CBAND	$-0.57^{+0.17}_{-0.12}$	$-1.21^{+0.04}_{-0.06}$	–	$1.93^{+0.12}_{-0.09}$	$2.86^{+0.04}_{-0.03}$	$1.54^{+0.15}_{-0.20}$	862/961
11.7	12.0	CPL	–	$-1.05^{+0.02}_{-0.01}$	–	–	$2.95^{+0.02}_{-0.03}$	$0.09^{+0.01}_{-0.01}$	935/963
12.0	12.2	CPL	–	$-1.04^{+0.03}_{-0.02}$	–	–	$2.96^{+0.03}_{-0.04}$	$0.06^{+0.01}_{-0.01}$	842/963
12.2	12.6	2SBPL	$-0.84^{+0.08}_{-0.03}$	$-1.47^{+0.07}_{-0.03}$	$-3.89^{+0.19}_{-0.94}$	$1.94^{+0.04}_{-0.10}$	$3.06^{+0.07}_{-0.03}$	$1.81^{+0.05}_{-0.13}$	910/960
12.6	12.9	2SBPL	$-0.74^{+0.14}_{-0.04}$	$-1.36^{+0.04}_{-0.05}$	$-4.11^{+0.33}_{-0.83}$	$1.79^{+0.08}_{-0.09}$	$3.00^{+0.05}_{-0.04}$	$1.70^{+0.06}_{-0.21}$	896/960

continued

Supplementary Data Table 2: Spectral fitting results and corresponding fitting statistics for GECAM S-I and S-II spectra. All errors represent the 1σ uncertainties.

t_1 (s)	t_2 (s)	Best model	α_1	$\alpha_2(\alpha)$	β	$\log E_\mathrm{b}$ (keV)	$\log E_\mathrm{p}(\log E_\mathrm{c})$ (keV)	$\log A$ (photons/cm^2/s/keV)	PGSTAT/dof
12.9	13.1	CSBPL	$-0.52^{+0.10}_{-0.15}$	$-1.15^{+0.04}_{-0.08}$	–	$1.64^{+0.14}_{-0.09}$	$2.85^{+0.03}_{-0.02}$	$1.56^{+0.21}_{-0.13}$	841/961
13.1	13.3	2SBPL	$-0.56^{+0.09}_{-0.11}$	$-1.39^{+0.03}_{-0.04}$	$-4.89^{+0.48}_{-0.76}$	$1.71^{+0.06}_{-0.06}$	$3.05^{+0.04}_{-0.04}$	$1.56^{+0.17}_{-0.13}$	860/960
13.3	13.5	2SBPL	$-0.87^{+0.04}_{-0.09}$	$-1.55^{+0.06}_{-0.06}$	$-5.07^{+1.10}_{-0.50}$	$1.93^{+0.10}_{-0.07}$	$3.17^{+0.04}_{-0.09}$	$1.96^{+0.14}_{-0.06}$	847/960
13.5	14.0	CBAND	$-0.31^{+0.06}_{-0.32}$	$-1.29^{+0.03}_{-0.04}$	–	$1.77^{+0.11}_{-0.03}$	$2.88^{+0.04}_{-0.03}$	$1.26^{+0.35}_{-0.07}$	885/961
14.0	14.4	2SBPL	$-0.63^{+0.22}_{-0.05}$	$-1.39^{+0.03}_{-0.02}$	$-4.17^{+0.58}_{-0.48}$	$1.61^{+0.03}_{-0.08}$	$3.02^{+0.04}_{-0.03}$	$1.59^{+0.08}_{-0.30}$	948/960
14.4	14.9	CBAND	$-0.55^{+0.06}_{-0.17}$	$-1.53^{+0.03}_{-0.06}$	–	$1.89^{+0.09}_{-0.03}$	$2.80^{+0.04}_{-0.04}$	$1.61^{+0.20}_{-0.08}$	884/961
14.9	15.3	CSBPL	$-0.57^{+0.22}_{-0.20}$	$-1.28^{+0.03}_{-0.07}$	–	$1.51^{+0.12}_{-0.08}$	$2.80^{+0.04}_{-0.03}$	$1.57^{+0.27}_{-0.29}$	817/961
15.3	15.9	CBAND	$-0.63^{+0.26}_{-0.10}$	$-1.46^{+0.05}_{-0.02}$	–	$1.84^{+0.04}_{-0.10}$	$2.94^{+0.02}_{-0.06}$	$1.71^{+0.12}_{-0.29}$	883/961
15.9	16.6	2SBPL	$-0.82^{+0.13}_{-0.08}$	$-1.46^{+0.02}_{-0.03}$	$-4.29^{+0.06}_{-1.20}$	$1.61^{+0.07}_{-0.07}$	$3.02^{+0.07}_{-0.01}$	$1.74^{+0.11}_{-0.18}$	869/960
16.6	17.4	CBAND	$-0.31^{+0.61}_{-0.23}$	$-1.47^{+0.04}_{-0.01}$	–	$1.67^{+0.03}_{-0.10}$	$2.83^{+0.03}_{-0.05}$	$1.32^{+0.25}_{-0.59}$	841/961
17.4	18.6	CPL	–	$-1.51^{+0.03}_{-0.03}$	–	–	$2.57^{+0.07}_{-0.06}$	$-0.55^{+0.02}_{-0.02}$	939/963
18.6	19.3	CBAND	$-0.06^{+0.42}_{-0.37}$	$-1.68^{+0.03}_{-0.04}$	–	$1.63^{+0.05}_{-0.05}$	$2.71^{+0.13}_{-0.06}$	$1.02^{+0.41}_{-0.41}$	871/961
19.3	19.9	CSBPL	$-0.90^{+0.10}_{-0.11}$	$-1.60^{+0.05}_{-0.05}$	–	$1.64^{+0.08}_{-0.07}$	$2.79^{+0.08}_{-0.04}$	$1.89^{+0.16}_{-0.14}$	900/961
19.9	20.4	CBAND	$-0.63^{+0.16}_{-0.13}$	$-1.52^{+0.03}_{-0.04}$	–	$1.83^{+0.07}_{-0.05}$	$3.07^{+0.04}_{-0.08}$	$1.69^{+0.15}_{-0.18}$	923/961
20.4	21.1	CBAND	$-0.81^{+0.54}_{-0.12}$	$-1.39^{+0.05}_{-0.02}$	–	$1.78^{+0.06}_{-0.19}$	$2.84^{+0.03}_{-0.05}$	$1.77^{+0.14}_{-0.56}$	930/961
21.1	21.5	CBAND	$-0.56^{+0.40}_{-0.20}$	$-1.38^{+0.03}_{-0.04}$	–	$1.76^{+0.08}_{-0.11}$	$2.87^{+0.07}_{-0.02}$	$1.58^{+0.24}_{-0.42}$	831/961
21.5	22.1	CBAND	$-0.87^{+0.16}_{-0.14}$	$-1.41^{+0.04}_{-0.04}$	–	$1.80^{+0.12}_{-0.08}$	$2.82^{+0.04}_{-0.04}$	$1.90^{+0.16}_{-0.19}$	893/961
22.1	22.9	2SBPL	$-0.83^{+0.11}_{-0.15}$	$-1.63^{+0.01}_{-0.04}$	$-5.60^{+1.31}_{-0.13}$	$1.54^{+0.07}_{-0.04}$	$2.97^{+0.03}_{-0.06}$	$1.70^{+0.20}_{-0.15}$	957/960
22.9	23.5	CBAND	$-0.88^{+0.12}_{-0.13}$	$-1.54^{+0.09}_{-0.12}$	–	$1.95^{+0.09}_{-0.10}$	$2.40^{+0.08}_{-0.03}$	$1.82^{+0.16}_{-0.13}$	846/961
23.5	24.3	CBAND	$-0.56^{+0.27}_{-0.12}$	$-1.68^{+0.04}_{-0.02}$	–	$1.75^{+0.03}_{-0.06}$	$2.73^{+0.07}_{-0.07}$	$1.60^{+0.14}_{-0.29}$	962/961
24.3	25.0	CBAND	$-1.08^{+0.08}_{-0.08}$	$-1.63^{+0.04}_{-0.08}$	–	$2.00^{+0.13}_{-0.07}$	$2.90^{+0.06}_{-0.08}$	$2.14^{+0.10}_{-0.11}$	779/961
25.0	25.6	CBAND	$-0.55^{+0.28}_{-0.23}$	$-1.61^{+0.07}_{-0.06}$	–	$1.74^{+0.09}_{-0.08}$	$2.42^{+0.05}_{-0.05}$	$1.62^{+0.25}_{-0.30}$	792/961
25.6	26.3	CPL	–	$-1.40^{+0.04}_{-0.03}$	–	–	$2.24^{+0.03}_{-0.03}$	$-0.20^{+0.03}_{-0.02}$	824/963
26.3	27.8	CBAND	$-0.70^{+0.21}_{-0.27}$	$-1.93^{+0.02}_{-0.04}$	–	$1.62^{+0.06}_{-0.03}$	$2.38^{+0.28}_{-0.11}$	$1.88^{+0.27}_{-0.21}$	945/961
27.8	28.5	CBAND	$-0.71^{+0.58}_{-0.08}$	$-1.77^{+0.03}_{-0.03}$	–	$1.70^{+0.02}_{-0.10}$	$2.96^{+0.22}_{-0.07}$	$1.86^{+0.09}_{-0.59}$	949/961
28.5	29.7	CPL	–	$-1.60^{+0.02}_{-0.02}$	–	–	$2.70^{+0.08}_{-0.07}$	$-0.58^{+0.01}_{-0.01}$	992/963
29.7	32.0	CPL	–	$-1.68^{+0.02}_{-0.02}$	–	–	$2.51^{+0.07}_{-0.06}$	$-0.71^{+0.01}_{-0.01}$	1027/963
32.0	33.3	CPL	–	$-1.83^{+0.03}_{-0.06}$	–	–	$2.03^{+0.11}_{-0.04}$	$-0.77^{+0.02}_{-0.04}$	911/963
33.3	34.0	CPL	–	$-1.78^{+0.02}_{-0.07}$	–	–	$2.45^{+0.53}_{-0.05}$	$-0.75^{+0.01}_{-0.04}$	926/963
34.0	34.7	CPL	–	$-1.65^{+0.04}_{-0.04}$	–	–	$2.29^{+0.09}_{-0.05}$	$-0.64^{+0.02}_{-0.03}$	864/963
34.7	35.6	CPL	–	$-1.68^{+0.03}_{-0.04}$	–	–	$2.41^{+0.12}_{-0.06}$	$-0.60^{+0.02}_{-0.02}$	897/963
35.6	36.8	CPL	–	$-1.61^{+0.05}_{-0.05}$	–	–	$2.11^{+0.05}_{-0.04}$	$-0.68^{+0.03}_{-0.03}$	935/963
36.8	37.5	CPL	–	$-1.65^{+0.04}_{-0.06}$	–	–	$2.19^{+0.09}_{-0.04}$	$-0.64^{+0.02}_{-0.04}$	884/963
37.5	38.9	CPL	–	$-1.64^{+0.04}_{-0.04}$	–	–	$2.23^{+0.06}_{-0.05}$	$-0.80^{+0.03}_{-0.03}$	937/963
38.9	40.6	CPL	–	$-1.83^{+0.02}_{-0.05}$	–	–	$2.32^{+0.29}_{-0.08}$	$-1.03^{+0.02}_{-0.03}$	895/963
40.6	44.6	CPL	–	$-1.95^{+0.03}_{-0.03}$	–	–	$1.69^{+0.11}_{-0.24}$	$-1.13^{+0.02}_{-0.02}$	1007/963
44.6	47.7	CPL	–	$-1.88^{+0.03}_{-0.04}$	–	–	$2.00^{+0.12}_{-0.07}$	$-1.14^{+0.02}_{-0.03}$	954/963
47.7	51.0	CPL	–	$-1.73^{+0.04}_{-0.05}$	–	–	$2.16^{+0.09}_{-0.05}$	$-1.08^{+0.02}_{-0.03}$	996/963
51.0	55.2	CPL	–	$-1.86^{+0.04}_{-0.06}$	–	–	$1.70^{+0.06}_{-0.11}$	$-1.24^{+0.03}_{-0.04}$	1016/963
55.2	61.6	CPL	–	$-1.94^{+0.06}_{-0.03}$	–	–	$1.35^{+0.19}_{-0.20}$	$-1.39^{+0.04}_{-0.02}$	979/963
61.6	69.6	CPL	–	$-1.94^{+0.04}_{-0.09}$	–	–	$(2.66^{+0.39}_{-0.10})^{*}$	$-1.55^{+0.03}_{-0.06}$	988/963
69.6	80.0	CPL	–	$-1.96^{+0.07}_{-0.15}$	–	–	$(2.42^{+0.52}_{-0.12})$	$-1.81^{+0.05}_{-0.12}$	944/963
80.0	90.0	PL	–	$-2.18^{+0.04}_{-0.05}$	–	–	–	$-2.05^{+0.02}_{-0.02}$	910/964
90.0	100.0	PL	–	$-2.33^{+0.08}_{-0.07}$	–	–	–	$-2.36^{+0.04}_{-0.04}$	908/964
100.0	115.0	PL	–	$-2.53^{+0.13}_{-0.19}$	–	–	–	$-2.86^{+0.08}_{-0.13}$	789/964
115.0	147.0	PL	–	$-3.30^{+0.27}_{-0.56}$	–	–	–	$-3.66^{+0.16}_{-0.42}$	542/964

* In cases where the 1σ lower limit of $\alpha_2(\alpha)$ nears or falls below -2, we use cutoff energy as a substitute for peak energy.

Supplementary Data Table 3: **Spectral fitting results and corresponding fitting statistics for LEIA S-III and GECAM S-III spectra.** All errors represent the 1σ uncertainties.

	LEIA S-III:*			GECAM S-III:						
Time (s)	Best model	α	CSTAT/dof	Best model	α_1	$\alpha_2(\alpha)$	β	$\log E_b$ (keV)	$\log E_p(\log E_c)$ (keV)	PGSTAT/dof
(1) 0–9	PL	$-1.34^{+0.19}_{-0.18}$	27/20	2SBPL	$-0.71^{+0.01}_{-0.01}$	$-1.15^{+0.03}_{-0.01}$	$-3.90^{+0.10}_{-0.03}$	$2.17^{+0.02}_{-0.05}$	$3.023^{+0.004}_{-0.012}$	1718/960
(2) 9–16	PL	$-1.24^{+0.18}_{-0.20}$	36/22	2SBPL	$-0.78^{+0.01}_{-0.03}$	$-1.37^{+0.01}_{-0.02}$	$-3.79^{+0.11}_{-0.09}$	$1.80^{+0.03}_{-0.02}$	$3.00^{+0.01}_{-0.01}$	1491/960
(3) 16–24	PL	$-1.37^{+0.19}_{-0.19}$	24/23	2SBPL	$-0.98^{+0.04}_{-0.02}$	$-1.65^{+0.01}_{-0.01}$	$-4.31^{+0.27}_{-0.42}$	$1.63^{+0.01}_{-0.03}$	$2.98^{+0.03}_{-0.02}$	1176/960
(4) 24–32	PL	$-1.65^{+0.18}_{-0.21}$	25/21	2SBPL	$-1.11^{+0.04}_{-0.06}$	$-1.81^{+0.01}_{-0.01}$	$-4.32^{+0.47}_{-1.04}$	$1.48^{+0.03}_{-0.02}$	$2.89^{+0.06}_{-0.05}$	1084/960
(5) 32–42	PL	$-1.53^{+0.17}_{-0.20}$	19/23	CPL	–	$-1.73^{+0.01}_{-0.02}$	–	–	$2.22^{+0.03}_{-0.02}$	1056/963
(6) 42–52	PL	$-1.91^{+0.19}_{-0.22}$	13/21	CPL	–	$-1.86^{+0.02}_{-0.03}$	–	–	$2.02^{+0.05}_{-0.04}$	1034/963
(7) 52–63	PL	$-1.51^{+0.17}_{-0.20}$	16/20	CPL	–	$-1.92^{+0.04}_{-0.04}$	–	–	$1.52^{+0.10}_{-0.17}$	1014/963
(8) 63–76	PL	$-1.46^{+0.18}_{-0.18}$	17/18	CPL	–	$-1.95^{+0.05}_{-0.05}$	–	–	$(2.66^{+0.20}_{-0.12})$†	889/963
(9) 76–91	PL	$-1.56^{+0.18}_{-0.22}$	18/20	CPL	–	$-2.03^{+0.09}_{-0.09}$	–	–	$(2.48^{+0.31}_{-0.18})$	886/963
(10) 91–147	PL	$-2.00^{+0.14}_{-0.16}$	23/21	CPL	–	$-2.51^{+0.30}_{-0.05}$	–	–	$(2.83^{+0.02}_{-0.76})$	399/963
(11) 147–243	PL	$-2.80^{+0.26}_{-0.27}$	26/19	–	–	–	–	–	–	–

	LEIA S-III & GECAM S-III:									
Time (s)	Best model	CPL.α	$PL_{sa}.\alpha_{sa}$	$PL_{sa}.\log E_{sa}$ (keV)	α_1	$\alpha_2(\alpha)$	β	$\log E_b$ (keV)	$\log E_p(\log E_c)$ (keV)	STAT/dof
(1) 0–9	CPL+PL_{sa}-2SBPL‡	$-1.37^{+0.14}_{-0.22}$	(1)	$1.13^{+0.03}_{-0.19}$	$-0.63^{+0.04}_{-0.04}$	$-1.12^{+0.03}_{-0.02}$	$-3.86^{+0.09}_{-0.03}$	$2.09^{+0.03}_{-0.04}$	$3.018^{+0.004}_{-0.011}$	$(27+1715)/977$**
(2) 9–16	CPL+PL_{sa}-2SBPL	$-1.50^{+0.11}_{-0.26}$	(1)	$1.16^{+0.01}_{-0.11}$	$-0.76^{+0.03}_{-0.02}$	$-1.37^{+0.01}_{-0.02}$	$-3.75^{+0.07}_{-0.13}$	$1.82^{+0.03}_{-0.02}$	$3.00^{+0.01}_{-0.01}$	$(38+1487)/979$
(3) 16–24	CPL+PL_{sa}-2SBPL	$-1.53^{+0.15}_{-0.20}$	(1)	$1.09^{+0.03}_{-0.21}$	$-0.93^{+0.05}_{-0.03}$	$-1.63^{+0.02}_{-0.01}$	$-4.18^{+0.20}_{-0.45}$	$1.64^{+0.02}_{-0.03}$	$2.97^{+0.03}_{-0.02}$	$(26+1176)/980$
(4) 24–32	CPL+PL_{sa}-2SBPL	$-1.83^{+0.20}_{-0.27}$	(1)	$1.00^{+0.08}_{-0.13}$	$-1.08^{+0.04}_{-0.07}$	$-1.81^{+0.02}_{-0.01}$	$-4.05^{+0.23}_{-1.25}$	$1.50^{+0.02}_{-0.03}$	$2.86^{+0.08}_{-0.02}$	$(25+1084)/978$
(5) 32–42	CPL+PL_{sa}-CPL	$-1.77^{+0.11}_{-0.24}$	(1.5)	$0.87^{+0.09}_{-0.05}$	–	$-1.70^{+0.02}_{-0.02}$	–	–	$(2.78^{+0.05}_{-0.04})$†	$(18+1055)/983$
(6) 42–52	CPL+PL_{sa}-CPL	$-2.19^{+0.26}_{-0.16}$	(1.5)	$0.84^{+0.10}_{-0.05}$	–	$-1.83^{+0.02}_{-0.03}$	–	–	$(2.83^{+0.11}_{-0.06})$	$(12+1034)/981$
(7) 52–63	CPL+PL_{sa}-CPL	$-1.89^{+0.21}_{-0.16}$	(1.5)	$0.80^{+0.10}_{-0.06}$	–	$-1.86^{+0.04}_{-0.05}$	–	–	$(2.59^{+0.17}_{-0.07})$	$(17+1015)/980$
(8) 63–76	CPL+PL_{sa}-CPL	$-1.83^{+0.13}_{-0.21}$	(1.5)	$0.69^{+0.16}_{-0.03}$	–	$-1.82^{+0.02}_{-0.11}$	–	–	$(2.60^{+0.35}_{-0.05})$	$(18+890)/978$
(9) 76–91	CPL+PL_{sa}-CPL	$-1.71^{+0.03}_{-0.33}$	(1.5)	$0.72^{+0.10}_{-0.07}$	–	$-1.93^{+0.06}_{-0.09}$	–	–	$(2.80^{+0.06}_{-0.32})$	$(18+887)/980$
(10) 91–147	CPL	$-1.71^{+0.04}_{-0.07}$				$\log E_p = 1.19^{+0.05}_{-0.05}$				$(26+403)/986$

* To align with the temporal divisions of the GECAM S-III spectra, we merge two bins, namely 91–113 s and 113–147 s, into a single bin. Consequently, there are 11 LEIA S-III spectra available for fitting the spectral energy distributions.

† In cases where the 1σ lower limit of $\alpha_2(\alpha)$ nears or falls below -2, we use cutoff energy as a substitute for peak energy.

‡ For the CPL components in the joint fittings, their cutoff energies are fixed at 53 keV to be same with that of the tenth SED's CPL model.

** In cases where LEIA and GECAM are jointly fitted, the reduced statistic is expressed as (CSTAT of LEIA + PGSTAT of GECAM)/dof.

Supplementary Data Table 4: Smoothly broken power law fitting results for the multi-wavelength flux light curves. All errors represent the 1σ uncertainties.

Energy (keV)	$\hat{\alpha}_1$	$\hat{\alpha}_2$	$t_{\rm b}$ (s)	$\log A$ (erg cm^{-2} s^{-1})	χ^2/dof
LF: 0.5–4	$0.40^{+0.05}_{-0.05}$	$2.32^{+0.16}_{-0.15}$	$79.6^{+5.5}_{-5.8}$	$-6.85^{+0.04}_{-0.04}$	12/17

Energy (keV)	$\hat{\alpha}_1$	$\hat{\alpha}_2$	$\hat{\alpha}_3$	$\hat{\alpha}_4$	$t_{\rm b1}$ (s)	$t_{\rm b2}$ (s)	$t_{\rm b3}$ (s)	$\log A$ (erg cm^{-2} s^{-1})	χ^2/dof
GF1: 15–30	$-0.70^{+0.04}_{-0.04}$	$0.11^{+0.02}_{-0.02}$	$2.45^{+0.02}_{-0.02}$	$5.2^{+0.4}_{-0.3}$	$7.5^{+0.4}_{-0.3}$	$26.3^{+0.2}_{-0.2}$		$-5.537^{+0.005}_{-0.005}$	
GF2: 30–100	$-0.76^{+0.01}_{-0.01}$	$0.47^{+0.01}_{-0.01}$	$2.85^{+0.01}_{-0.01}$	$6.9^{+0.5}_{-0.4}$	$6.47^{+0.06}_{-0.06}$	$22.0^{+0.1}_{-0.1}$	$84.3^{+1.2}_{-1.2}$	$-4.755^{+0.002}_{-0.002}$	40864/249
GF3: 100–350	$-0.46^{+0.01}_{-0.01}$	$1.30^{+0.01}_{-0.01}$	$3.34^{+0.02}_{-0.02}$	$8.8^{+0.9}_{-0.8}$	$6.75^{+0.03}_{-0.03}$	$21.0^{+0.1}_{-0.1}$		$-4.104^{+0.002}_{-0.002}$	
GF4: 350–700	$-0.37^{+0.01}_{-0.01}$	$1.89^{+0.01}_{-0.01}$	$4.12^{+0.06}_{-0.06}$	> 6.5	$6.73^{+0.03}_{-0.03}$	$22.0^{+0.2}_{-0.2}$	(84.3)	$-3.976^{+0.002}_{-0.002}$	17452/86
GF5: 700–2000	$-0.24^{+0.02}_{-0.02}$	$2.24^{+0.04}_{-0.04}$	$5.3^{+0.3}_{-0.3}$	–	$6.69^{+0.05}_{-0.04}$	$22.0^{+1.3}_{-1.4}$	–	$-3.636^{+0.004}_{-0.004}$	8638/87

References

[1] Xiong S, Wang C, Huang Y et al. GRB 230307A: GECAM detection of an extremely bright burst. GRB Coordinates Network 2023; **33406**: 1.

[2] Fermi GBM Team. GRB 230307A: Fermi GBM detection of a very bright GRB. GRB Coordinates Network 2023; **33407**: 1.

[3] Fermi GBM Team. GRB 230307A: possibly the second highest GRB energy fluence ever identified. GRB Coordinates Network 2023; **33414**: 1.

[4] Svinkin D, Frederiks D, Ulanov M et al. Konus-Wind detection of GRB 230307A. GRB Coordinates Network 2023; **33427**: 1.

[5] Kozyrev AS, Golovin DV, Litvak ML et al. Further improved IPN localization for GRB 230307A. GRB Coordinates Network 2023; **33461**: 1.

[6] Evans PA and Swift Team. GRB 230307A: Tiled Swift observations. GRB Coordinates Network 2023; **33419**: 1.

[7] Burrows DN, Gropp JD, Osborne JP et al. GRB 230307A: Swift-XRT afterglow detection. GRB Coordinates Network 2023; **33465**: 1.

[8] Liu MJ, Wang YL, Liu Y et al. GRB 230307A: soft X-ray detection with LEIA. GRB Coordinates Network 2023; **33466**: 1.

[9] O'Connor B, Dichiara S, Troja E et al. GRB 230307A: Gemini-South Confirmation of the Optical Afterglow. GRB Coordinates Network 2023; **33447**: 1.

[10] Gillanders J, O'Connor B, Dichiara S et al. GRB 230307A: Continued Gemini-South observations confirm rapid optical fading. GRB Coordinates Network 2023; **33485**: 1.

[11] Levan AJ, Gompertz BP, Malesani DB et al. GRB 230307A: JWST observations consistent with the presence of a kilonova. GRB Coordinates Network 2023; **33569**: 1.

[12] Levan AJ, Watson D, Hjorth J et al. GRB 230307A: JWST NIRSpec observations, possible higher redshift. GRB Coordinates Network 2023; **33580**: 1.

[13] Levan AJ, Tanvir NR, Burns E et al. GRB 230307A: JWST second-epoch observations. GRB Coordinates Network 2023; **33747**: 1.

[14] Levan A, Gompertz BP, Salafia OS et al. JWST detection of heavy neutron capture elements in a compact object merger. arXiv e-prints 2023; arXiv:2307.02098.

[15] Zhang C, Ling ZX, Sun XJ et al. First Wide Field-of-view X-Ray Observations by a Lobster-eye Focusing Telescope in Orbit. Astrophys. J. Let. 2022; **941**:L2.

[16] Ling ZX, Sun XJ, Zhang C et al. The Lobster Eye Imager for Astronomy Onboard the SATech-01 Satellite. arXiv e-prints 2023; arXiv:2305.14895.

[17] Cheng H, Ling Z, Zhang C et al. Ground Calibration Result of the Lobster Eye Imager for Astronomy. arXiv e-prints 2023; arXiv:2312.06964.

[18] Rukdee S, Burwitz V, Hartner G et al. The X-ray testing of Einstein Probe Wide-field X-ray Telescope Qualification Model at PANTER. Minoglou K, Karafolas N and Cugny B, editors, Society of Photo-Optical Instrumentation Engineers (SPIE) Conference Series, volume 12777 of Society of Photo-Optical Instrumentation Engineers (SPIE) Conference Series (2023) 127773F.

[19] Li XQ, Wen XY, An ZH et al. The technology for detection of gamma-ray burst with GECAM satellite. Radiation Detection Technology and Methods 2021; **6**: 12–25.

[20] Zhang D, Zheng C, Liu J et al. The performance of SiPM-based gamma-ray detector (GRD) of GECAM-C. arXiv e-prints 2023; arXiv:2303.00537.

[21] Zhao XY, Xiong SL, Wen XY et al. The In-Flight Realtime Trigger and Localization Software of GECAM. arXiv e-prints 2021; arXiv:2112.05101.

[22] Guo S, Li G, Zheng J et al. Integrated Navigation and Communication Service for LEO Satellites Based on BDS-3 Global Short Message Communication. IEEE Access 2023; **11**: 6623–6631.

[23] An Z, Sun X, Dali Z et al. The design and performance of GRD onboard the GECAM satellite. Radiation Detection Technology and Methods 2021; **6**: 43–52.

[24] Zheng C, Peng WX, Li XB et al. Electron non-linear light yield of $LaBr_3$ detector aboard GECAM. Nuclear Instruments and Methods in Physics Research A 2022; **1042**:167427.

[25] Zheng C, An ZH, Peng WX et al. Ground calibration of Gamma-Ray Detectors of GECAM-C. arXiv e-prints 2023; arXiv:2303.00687.

[26] Zhang YQ, Xiong SL, Qiao R et al. Cross calibration of gamma-ray detectors (GRD) of GECAM-C. arXiv e-prints 2023; arXiv:2303.00698.

[27] Xu YB, Sun XL, Yang S et al. The design and performance of charged particle detector onboard the GECAM mission. Radiation Detection Technology and Methods 2023; **6**: 53–62.

[28] Huang Y, Shi D, Zhang X et al. The GECAM Real-Time Burst Alert System. arXiv e-prints 2023; arXiv:2307.04999.

[29] Fermi GBM Team. GRB 230307A: Bad Time Intervals for Fermi GBM data. GRB Coordinates Network 2023; **33551**: 1.

[30] Liu YQ, Gong K, Li XQ et al. The SiPM Array Data Acquisition Algorithm Applied to the GECAM Satellite Payload. arXiv e-prints 2021; arXiv:2112.04786.

[31] Lü HJ, Zhang B, Liang EW et al. The 'amplitude' parameter of gamma-ray bursts and its implications for GRB classification. Mon. Not. R. Astron. Soc. 2014; **442**: 1922–1929.

[32] Golkhou VZ, Butler NR and Littlejohns OM. The Energy Dependence of GRB Minimum Variability Timescales. Astrophys. J. 2015; **811**:93.

[33] Camisasca AE, Guidorzi C, Amati L et al. GRB minimum variability timescale with Insight-HXMT and Swift. Implications for progenitor models, dissipation physics, and GRB classifications. Astron. Astrophys. 2023; **671**:A112.

[34] MacLachlan GA, Shenoy A, Sonbas E et al. Minimum variability time-scales of long and short GRBs. Mon. Not. R. Astron. Soc. 2013; **432**: 857–865.

[35] Scargle JD, Norris JP, Jackson B et al. Studies in Astronomical Time Series Analysis. VI. Bayesian Block Representations. Astrophys. J. 2013; **764**:167.

[36] Yi T, Liang E, Qin Y et al. On the spectral lags of the short gamma-ray bursts. Mon. Not. R. Astron. Soc. 2006; **367**: 1751–1756.

[37] Bernardini MG, Ghirlanda G, Campana S et al. Comparing the spectral lag of short and long gamma-ray bursts and its relation with the luminosity. Mon. Not. R. Astron. Soc. 2015; **446**: 1129–1138.

[38] Norris JP, Marani GF and Bonnell JT. Connection between Energy-dependent Lags and Peak Luminosity in Gamma-Ray Bursts. Astrophys. J. 2000; **534**: 248–257.

[39] Ukwatta TN, Dhuga KS, Stamatikos M et al. The lag-luminosity relation in the GRB source frame: an investigation with Swift BAT bursts. Mon. Not. R. Astron. Soc. 2012; **419**: 614–623.

[40] Zhang BB, Burrows DN, Zhang B et al. Unusual Central Engine Activity in the Double Burst GRB 110709B. Astrophys. J. 2012; **748**:132.

[41] Arnaud KA. XSPEC: The First Ten Years. Jacoby GH and Barnes J, editors, Astronomical Data Analysis Software and Systems V, volume 101 of Astronomical Society of the Pacific Conference Series (1996) 17.

[42] Wilms J, Allen A and McCray R. On the Absorption of X-Rays in the Interstellar Medium. Astrophys. J. 2000; **542**: 914–924.

[43] Cash W. Parameter estimation in astronomy through application of the likelihood ratio. Astrophys. J. 1979; **228**: 939–947.

[44] Willingale R, Starling RLC, Beardmore AP et al. Calibration of X-ray absorption in our Galaxy. Mon. Not. R. Astron. Soc. 2013; **431**: 394–404.

[45] Mereghetti S, Rigoselli M, Salvaterra R et al. XMM-Newton and INTEGRAL Observations of the Bright GRB 230307A: Vanishing of the Local Absorption and Limits on the Dust in the Magellanic Bridge. Astrophys. J. 2023; **956**:97.

[46] Campana S, Lazzati D, Perna R et al. The variable absorption in the X-ray spectrum of GRB 190114C. Astron. Astrophys. 2021; **649**:A135.

[47] Yang J, Ai S, Zhang BB et al. A long-duration gamma-ray burst with a peculiar origin. Nature 2022; **612**: 232–235.

[48] Yang J, Zhao XH, Yan Z et al. Synchrotron Radiation Dominates the Extremely Bright GRB 221009A. Astrophys. J. Let. 2023; **947**:L11.

[49] Feroz F and Hobson MP. Multimodal nested sampling: an efficient and robust alternative to Markov Chain Monte Carlo methods for astronomical data analyses. Mon. Not. R. Astron. Soc. 2008; **384**: 449–463.

[50] Feroz F, Hobson MP and Bridges M. MULTINEST: an efficient and robust Bayesian inference tool for cosmology and particle physics. Mon. Not. R. Astron. Soc. 2009; **398**: 1601–1614.

[51] Buchner J, Georgakakis A, Nandra K et al. X-ray spectral modelling of the AGN obscuring region in the CDFS: Bayesian model selection and catalogue. Astron. Astrophys. 2014; **564**:A125.

[52] Feroz F, Hobson MP, Cameron E et al. Importance Nested Sampling and the MultiNest Algorithm. The Open Journal of Astrophysics 2019; **2**:10.

[53] Band D, Matteson J, Ford L et al. BATSE Observations of Gamma-Ray Burst Spectra. I. Spectral Diversity. Astrophys. J. 1993; **413**: 281.

[54] Ravasio ME, Oganesyan G, Ghirlanda G et al. Consistency with synchrotron emission in the bright GRB 160625B observed by Fermi. Astron. Astrophys. 2018; **613**:A16.

[55] Zheng W, Shen RF, Sakamoto T et al. Panchromatic Observations of the Textbook GRB 110205A: Constraining Physical Mechanisms of Prompt Emission and Afterglow. Astrophys. J. 2012; **751**:90.

[56] Schwarz G. Estimating the Dimension of a Model. Annals of Statistics 1978; **6**: 461–464.

[57] Jeffreys H. Theory of Probability, (OuP Oxford1939).

[58] Kass RE and Raftery AE. Bayes factors. Journal of the american statistical association 1995; **90**: 773–795.

[59] Mukherjee S, Feigelson ED, Jogesh Babu G et al. Three Types of Gamma-Ray Bursts. Astrophys. J. 1998; **508**: 314–327.

[60] Zhang BB, Zhang B, Castro-Tirado AJ et al. Transition from fireball to Poynting-flux-dominated outflow in the three-episode GRB 160625B. Nature Astronomy 2018; **2**: 69–75.

[61] Amati L, Frontera F, Tavani M et al. Intrinsic spectra and energetics of BeppoSAX Gamma-Ray Bursts with known redshifts. Astron. Astrophys. 2002; **390**: 81–89.

[62] Zhang B, Zhang BB, Virgili FJ et al. Discerning the Physical Origins of Cosmological Gamma-ray Bursts Based on Multiple Observational Criteria: The Cases of z = 6.7 GRB 080913, z = 8.2 GRB 090423, and Some Short/Hard GRBs. Astrophys. J. 2009; **703**: 1696–1724.

[63] Minaev PY and Pozanenko AS. The $E_{p,I}$-E_{iso} correlation: type I gamma-ray bursts and the new classification method. Mon. Not. R. Astron. Soc. 2020; **492**: 1919–1936.

[64] Foreman-Mackey D, Hogg DW, Lang D et al. emcee: The MCMC Hammer. Publ. Astron. Soc. Pac. 2013; **125**: 306.

[65] Lelli F, McGaugh SS, Schombert JM et al. The baryonic Tully-Fisher relation for different velocity definitions and implications for galaxy angular momentum. Mon. Not. R. Astron. Soc. 2019; **484**: 3267–3278.

[66] An ZH, Antier S, Bi XZ et al. Insight-HXMT and GECAM-C observations of the brightest-of-all-time GRB 221009A. arXiv e-prints 2023; arXiv:2303.01203.

[67] Ukwatta TN, Stamatikos M, Dhuga KS et al. Spectral Lags and the Lag-Luminosity Relation: An Investigation with Swift BAT Gamma-ray Bursts. Astrophys. J. 2010; **711**: 1073–1086.

[68] Xiao S, Xiong SL, Wang Y et al. A Robust Estimation of Lorentz Invariance Violation and Intrinsic Spectral Lag of Short Gamma-Ray Bursts. Astrophys. J. Let. 2022; **924**:L29.

[69] Yi SX, Wang CW, Zhang B et al. Evidence of mini-jet emission in a large emission zone from a magnetically-dominated gamma-ray burst jet. arXiv e-prints 2023; arXiv:2310.07205.

[70] Kumar P and Panaitescu A. Afterglow Emission from Naked Gamma-Ray Bursts. Astrophys. J. Let. 2000; **541**: L51–L54.

[71] Dermer CD. Curvature Effects in Gamma-Ray Burst Colliding Shells. Astrophys. J. 2004; **614**: 284–292.

[72] Zhang B, Fan YZ, Dyks J et al. Physical Processes Shaping Gamma-Ray Burst X-Ray Afterglow Light Curves: Theoretical Implications from the Swift X-Ray Telescope Observations. Astrophys. J. 2006; **642**: 354–370.

[73] Liang EW, Zhang B, O'Brien PT et al. Testing the Curvature Effect and Internal Origin of Gamma-Ray Burst Prompt Emissions and X-Ray Flares with Swift Data. Astrophys. J. 2006; **646**: 351–357.

[74] Uhm ZL and Zhang B. On the Curvature Effect of a Relativistic Spherical Shell. Astrophys. J. 2015; **808**:33.

[75] Zhang BB, Liang EW and Zhang B. A Comprehensive Analysis of Swift XRT Data. I. Apparent Spectral Evolution of Gamma-Ray Burst X-Ray Tails. Astrophys. J. 2007; **666**: 1002–1011.

[76] Zhang BB, Zhang B, Liang EW et al. Curvature Effect of a Non-Power-Law Spectrum and Spectral Evolution of GRB X-Ray Tails. Astrophys. J. Let. 2009; **690**: L10–L13.

[77] Zhang B and Yan H. The Internal-collision-induced Magnetic Reconnection and Turbulence (ICMART) Model of Gamma-ray Bursts. Astrophys. J. 2011; **726**:90.

[78] Zhang B and Zhang B. Gamma-Ray Burst Prompt Emission Light Curves and Power Density Spectra in the ICMART Model. Astrophys. J. 2014; **782**:92.

[79] Shen RF and Zhang B. Prompt optical emission and synchrotron self-absorption constraints on emission site of GRBs. Mon. Not. R. Astron. Soc. 2009; **398**: 1936–1950.

[80] Yan ZY. et al. in preparation 2024; .

[81] Yang YH, Troja E, O'Connor B et al. A lanthanide-rich kilonova in the aftermath of a long gamma-ray burst. Nature 2024; **626**: 742–745.

[82] Dai CY, Guo CL, Zhang HM et al. Evidence for a Compact Stellar Merger Origin for GRB 230307A From Fermi-LAT and Multiwavelength Afterglow Observations. Astrophys. J. Let. 2024; **962**:L37.

[83] Wang XI, Yu YW, Ren J et al. What Powered the Kilonova-like Emission after GRB 230307A in the Framework of a Neutron Star–White Dwarf Merger? Astrophys. J. Let. 2024; **964**:L9.

[84] Bloom JS, Kulkarni SR and Djorgovski SG. The Observed Offset Distribution of Gamma-Ray Bursts from Their Host Galaxies: A Robust Clue to the Nature of the Progenitors. Astron. J. 2002; **123**: 1111–1148.

[85] Corwin J Harold G, Buta RJ and de Vaucouleurs G. Corrections and additions to the Third Reference Catalogue of Bright Galaxies. Astron. J. 1994; **108**: 2128–2144.

[86] Dey A, Schlegel DJ, Lang D et al. Overview of the DESI Legacy Imaging Surveys. Astron. J. 2019; **157**:168.

[87] Fausnaugh MM, Jayaraman R, Vanderspek R et al. Observations of GRB 230307A by TESS. Research Notes of the American Astronomical Society 2023; **7**:56.

[88] Shapiro SL and Teukolsky SA. Black holes, white dwarfs and neutron stars. The physics of compact objects (1983).

[89] Usov VV. Millisecond pulsars with extremely strong magnetic fields as a cosmological source of γ-ray bursts. Nature 1992; **357**: 472–474.

[90] Zhang B and Mészáros P. Gamma-Ray Burst Afterglow with Continuous Energy Injection: Signature of a Highly Magnetized Millisecond Pulsar. Astrophys. J. Let. 2001; **552**: L35–L38.

[91] Gao H, Zhang B and Lü HJ. Constraints on binary neutron star merger product from short GRB observations. Phys. Rev. D 2016; **93**:044065.

[92] Sun H, Li Y, Zhang BB et al. A Unified Binary Neutron Star Merger Magnetar Model for the Chandra X-Ray Transients CDF-S XT1 and XT2. Astrophys. J. 2019; **886**:129.

[93] Lasky PD, Haskell B, Ravi V et al. Nuclear equation of state from observations of short gamma-ray burst remnants. Phys. Rev. D 2014; **89**:047302.

[94] Evans PA, Beardmore AP, Page KL et al. An online repository of Swift/XRT light curves of γ-ray bursts. Astron. Astrophys. 2007; **469**: 379–385.

[95] Evans PA, Beardmore AP, Page KL et al. Methods and results of an automatic analysis of a complete sample of Swift-XRT observations of GRBs. Mon. Not. R. Astron. Soc. 2009; **397**: 1177–1201.

[96] Evans PA, Willingale R, Osborne JP et al. The Swift Burst Analyser. I. BAT and XRT spectral and flux evolution of gamma ray bursts. Astron. Astrophys. 2010; **519**:A102.

[97] Xue YQ, Zheng XC, Li Y et al. A magnetar-powered X-ray transient as the aftermath of a binary neutron-star merger. Nature 2019; **568**: 198–201.

[98] Yu YW, Zhang B and Gao H. Bright "Merger-nova" from the Remnant of a Neutron Star Binary Merger: A Signature of a Newly Born, Massive, Millisecond Magnetar. Astrophys. J. Let. 2013; **776**:L40.

[99] Ai S, Zhang B and Zhu Z. Engine-fed kilonovae (mergernovae) - I. Dynamical evolution and energy injection/heating efficiencies. Mon. Not. R. Astron. Soc. 2022; **516**: 2614–2628.

[100] Wang Y, Zhang B and Zhu Z. Anisotropic Energy Injection from Magnetar Central Engines in Short GRBs. arXiv e-prints 2023; arXiv:2309.15141.

[101] Gillanders JH, Troja E, Fryer CL et al. Heavy element nucleosynthesis associated with a gamma-ray burst. arXiv e-prints 2023; arXiv:2308.00633.

[102] Lu W and Quataert E. Late-time accretion in neutron star mergers: Implications for short gamma-ray bursts and kilonovae. Mon. Not. R. Astron. Soc. 2023; **522**: 5848–5861.

[103] Kisaka S and Ioka K. Long-lasting Black Hole Jets in Short Gamma-Ray Bursts. Astrophys. J. Let. 2015; **804**:L16.

[104] Li Y, Zhang B and Lü HJ. A Comparative Study of Long and Short GRBs. I. Overlapping Properties. Astrophys. J. Supp. 2016; **227**:7.
